# The role of N-cadherin/c-Jun/NDRG1 axis in the progression of prostate cancer

**DOI:** 10.7150/ijbs.63300

**Published:** 2021-07-25

**Authors:** Yongjun Quan, Xiaodong Zhang, William Butler, Zhen Du, Mingdong Wang, Yuexin Liu, Hao Ping

**Affiliations:** 1 Department of Urology, Beijing Tongren Hospital, Capital Medical University, Beijing 100730, China.; 2 Department of Urology, Beijing Chaoyang Hospital, Capital Medical University, Beijing 100020, China.; 3 Department of Pathology, Duke University School of Medicine, Durham NC 27710, USA.

**Keywords:** prostate cancer, N-cadherin, androgen receptor, NDRG1, c-Jun, DNA methylation, enzalutamide

## Abstract

The dysregulation of androgen receptor (*AR*) signaling is a critical event in the progression of prostate cancer (PCa) and hormone therapy consisting of androgen deprivation (ADT) or AR inhibition is therefore used to treat advanced cases. It is known that N-cadherin becomes upregulated following ADT and can directly induce PCa transformation to the castration-resistant stage (CRPC). However, the relationship between AR and N-cadherin is unclear and may promote better understanding of CRPC pathogenesis and progression. Here, we demonstrate a new axis of N-cadherin/c-Jun/N-myc downstream regulated gene 1 (*NDRG1*) that N-cadherin promotes c-Jun expression and suppresses NDRG1 to promote invasion and migration of PCa cells through epithelial to mesenchymal transition (EMT). Targeting N-cadherin in combination with enzalutamide (ENZ) treatment synergistically suppressed PC3 cell proliferation *in vivo* and *in vitro*. Further studies showed that compared to lower Gleason score (GS) (GS < 7) cases, high GS (GS > 7) cases exhibited elevated N-cadherin expression and reduced NDRG1 expression, corroborating our *in vitro* observations. We further demonstrate that c-Jun, AR, and DNA methyltransferase-1 (*DNMT1*) form a complex in the 12-O-tetradecanoyl phorbol-13-acetate (TPA) response elements (TREs) region of the NDRG1 promoter, which suppresses NDRG1 transcription through DNA hypermethylation. In conclusion, we demonstrate an underlying mechanism for how N-cadherin collaborates with AR and NDRG1 to promote CRPC progression. Controlling N-cadherin/c-Jun/NDRG1 axis may help to overcome resistance to commonly used hormone therapy to improve long-term patient outcomes.

## Introduction

Prostate Cancer (PCa) is a common malignancy and leading cause of cancer-related mortality in men over the age of 50. In 2019, there were 174,650 new cases reported along with 31,620 deaths, indicating that PCa remains a serious health concern for older men and that more research is needed to ultimately improve long-term outcomes 1. Although most cases of PCa are diagnosed early and treated with local intervention, advanced cases are treated primarily with androgen deprivation therapy (ADT), as most PCa cells express high levels of androgen receptor (*AR*) which is critical to their survival. However, after a period of treatment, the cancer becomes resistant to hormone therapy, which is termed castration-resistant PCa (CRPC). CRPC is more aggressive than hormone-sensitive PCa and is associated with poor survival rates 2.

Elucidating the mechanisms underlying transformation to CRPC is extremely crucial for preventing treatment resistance and improving long-term patient outcomes. Dysregulation of the AR signaling axis is well known to be a critical event in both the development and pathogenesis of CRPC. AR has been reported to perform dual effects on PCa progression 3-8. For example, AR is well known to promote proliferation in PCa and inhibition of the protein suppresses tumor growth. However, studies have increasingly demonstrated that inhibition of AR activity is a major cause of the emergence of compensation mechanisms which lead to the much more lethal CRPC 6-19. Furthermore, low AR signaling has been shown to induce neuroendocrine PCa (NEPC) transformation 20-24 which is associated with a poor clinical prognosis 25.

Our previous study suggested that reduced AR/N-myc downstream regulated gene 1 (*NDRG1*) signaling may play an important role in PCa progression 26. As an iron-regulated metastasis suppressor 27, 28, NDRG1 has also been reported to perform antimetastatic functions in PCa 26, 29-31. By correlating IHC data with patient survival, it was observed that decreased membranous expression of NDRG1 was commonly observed in lower disease-free survival rates, and that the expression of NDRG1 was positively correlated with the expression of E-cadherin 29. NDRG1 is transcriptionally regulated by AR 32, 33 and inhibits the expression of mesenchymal markers (N-cadherin, Vimentin, Slug, Snail, etc.) 28, 34-36. The promoter of NDRG1 contains an androgen response element (ARE) at position -984, and AR binds this ARE to promote transcription 32, 33. Therefore, the expression of NDRG1 is directly induced by dihydrotestosterone (DHT) 37, 38 and methyltrienolone (R1881) 39, 40 treatment. Our previous study found that histone methylation plays an important role in AR-induced NDRG1 transcriptional activity 26.

N-cadherin, also called Cadherin-2 (*CDH2*), is a transmembrane protein that mediates cell-cell adhesion and cell migration 41. N-cadherin promotes cell migration and invasion and is an important factor in the epithelial to mesenchymal transition (EMT) 10, 42, 43. After inhibition of AR signaling, the expression of N-cadherin was significantly upregulated and directly induced PCa progression 10, 12, 17, 18, 44. As previously reported, DHT and R1881 treatment could also suppress the expression of N-cadherin 10, 45.

In our present study, N-cadherin in PCa cells significantly promoted the expression of c-Jun and suppressed the expression of AR and NDRG1. N-cadherin was previously reported to be suppressed by NDRG1 34, 35. Thus, NDRG1 not only is an upstream regulatory gene but also could interplay with N-cadherin. As a basic leucine zipper (bZIP) transcription factor, c-Jun can homodimerize or heterodimerize with c-Fos to form activator protein-1 (AP-1) and bind to the 12-O-tetradecanoyl phorbol-13-acetate (TPA) response element (TRE) to regulate target genes 46. The relationship between c-Jun and AR in terms of their transcriptional activity is controversial, and their functions as both coactivators 47, 48 and corepressors 49-58 have been reported. However, an important function of c-Jun is its ability to induce DNA methylation 59-61 by binding to the promoter of DNA methyltransferase-1 (DNMT1) 60, 62 or directly forming a complex with DNMT1 61.

Considering that N-cadherin affects the relative expression of NDRG1 and that two TREs have been found in the NDRG1 promoter, we hypothesized that c-Jun-mediated repression of NDRG1 transcription may play a crucial role in the AR/NDRG1/N-cadherin axis. Increased N-cadherin may inhibit AR/NDRG1 signaling through c-Jun, and NDRG1 suppression could further promote the expression of N-cadherin, leading to a vicious cycle and progression of CRPC. Controlling this axis through N-cadherin may restore the transcription of NDRG1 and ultimately reverse CRPC progression.

In this study, we evaluated the molecular mechanism of CRPC progression and attempted to further reverse CRPC by endogenously restoring NDRG1 transcription through controlling the N-cadherin/c-Jun/NDRG1 axis.

## Materials and methods

### Cell culture

The LNCaP cell line was used as an androgen-dependent cell line, and the PC3 cell line was used as an androgen-independent cell line (American Type Culture Collection, Rockville, MD, USA). Cells were cultured in RPMI-1640 medium supplemented with 10% fetal bovine serum (FBS) (HyClone, South Logan, UT, USA) and 1% antibiotic-antimycotic (AA) (Gibco, Grand Island, NY, USA). Cells were cultured in a humidified atmosphere at 37°C and 5% CO_2_.

### Gene regulation in PCa cell lines

To stably overexpress/downregulate specific genes, lentiviruses harboring specific gene vectors or specific short hairpin RNA (shRNA) sequences were generated by GENECHEM (Shanghai, China). PCa cells were infected with the lentiviruses with 5 μg/ml polybrene for 24 hours (h), and the medium was changed. After 72 h, the successfully transduced cells were selected with 1-2 μg/ml puromycin.

To transiently knockdown the expression of specific genes, small interfering RNAs (siRNAs) (GenePharma, Shanghai, China) specific to these genes were transfected into PCa cells using Lipofectamine 3000 (Invitrogen, Carlsbad, CA, USA) according to the manufacturer's instructions. Information regarding these genes is provided in Supplementary Table 1.

### Cell viability assay

In total, 2 × 10^3^ cells were seeded in 96-well culture dishes and cultured for 48-96 h. At the detection time point, the supernatant was discarded, medium containing 10% Cell Counting Kit-8 (CCK-8) (MedChemExpress (MCE), Monmouth Junction, NJ, USA) solution was added, and the sample was incubated (37°C) for 1 h. The absorbance at 450 nm in each well was measured through Varioskan Flash (Thermo Fisher Scientific, Waltham, MA, USA).

### Colony formation assay

Cells (3 × 10^3^ for LNCaP and 2 × 10^3^ for PC3) were evenly seeded in 6-well plates and cultured for 15 days to form visible colonies. Then, the cells were fixed (with 10% neutral buffered formalin solution) and stained with 0.01% crystal violet solution (Beyotime, Shanghai, China). The cells were fully decolorized, and the number of colonies was counted.

### Transwell invasion assay

As previously described 26, Transwell chambers (Corning, NY, USA) were used to evaluate cell invasion. Cells (5 × 10^4^) in RPMI medium (serum-free) were seeded into the upper chamber with an insert coated with Matrigel (BD Biosciences, San Jose, CA, USA). The lower chamber was filled with 500 μl medium with 20% FBS as a chemoattractant. After 48 h of incubation, the cells on the upper surface of the Transwell membranes were removed, and the membranes were fixed (10% neutral buffered formalin solution), stained (0.01% crystal violet solution (Beyotime)), and counted.

### Wound healing assay

Cell migration was evaluated through a wound healing assay as previously described 26. Cells (5 × 10^5^) at more than 90% confluence were seeded in 6-well plates. A scratch was generated in the middle of the wells using a 200-μl pipette tip, and the cells were cultured for 48 h. Cell migration was imaged by light microscopy at 0 h, 24 h, and 48 h. The cell-free areas were analyzed with ImageJ software (National Institutes of Health (NIH), Bethesda, MD, USA).

### Reverse transcription (RT) and quantitative real-time PCR (qPCR) analysis

TRIzol™ reagent (Invitrogen) was used to isolate total RNA. Complementary DNA (cDNA) was synthesized through One-Step gDNA Removal and cDNA Synthesis SuperMix (TransGen Biotech, Beijing, China) with anchored oligo (dT) primers according to the manufacturer's instructions. qPCR was performed using Top Green qPCR SuperMix (TransGen Biotech) on an SDS 7500 FAST Real-Time PCR system (Applied Biosystems, Foster City, CA, USA). The endogenous reference gene used for normalization was GAPDH or 18S ribosomal RNA. The relevant primer sequences are shown in Supplementary Table 2.

### Western blot (WB) analysis

WB analysis was performed as previously described 26. Total protein was extracted using radioimmunoprecipitation assay (RIPA) buffer (Solarbio, Beijing, China) and quantified using a BCA Protein Assay Kit (KeyGen Biotech, Nanjing, China). The proteins were separated with SDS-polyacrylamide gel electrophoresis (SDS-PAGE) and transferred to polyvinylidene difluoride (PVDF) membranes (Merck Millipore, Billerica, MA, USA). The membranes were blocked in 5% skim milk for 1 h, incubated with primary antibodies at 4°C overnight and then incubated with secondary antibodies at room temperature for 1 h. Chemiluminescent HRP Substrate (Merck Millipore) and ChemiDoc™ XRS+ with Image Lab™ software (Bio-Rad, Hercules, CA, USA) were used to detect the immunoreactive bands. Information regarding the antibodies is provided in Supplementary Table 3.

### Co-immunoprecipitation (Co-IP) assay

Co-IP assay was performed as previously described 26. Cells were lysed in lysis buffer (R0030, Solarbio) and incubated with 5 μg of anti-cJun or same-host-sourced anti-IgG antibody at 4°C overnight. On the following day, protein A/G agarose beads (GE Healthcare, Little Chalfont, Buckinghamshire, UK) were added to the lysis buffer and then incubated at 4°C for 2 h. Then, immune complexes were eluted in denaturing SDS sample buffer, and a WB (immunoblotting) analysis was performed with anti-AR, anti-DNMT1, and anti-cJun antibodies. Information regarding the relevant antibodies is provided in Supplementary Table 4.

### Chromatin immunoprecipitation (ChIP) assay

An EZ-ChIP kit (Cat# 17-371, Merck Millipore) was used to perform the ChIP assay according to the manufacturer's instructions. Information regarding the antibodies used for immunoprecipitation is provided in Supplementary Table 4. The levels of immunoprecipitated genes were evaluated through PCR and qPCR. The related oligonucleotide primers for NDRG1 containing TRE (1), ARE, and TRE (2) sequences are listed in Supplementary Table 5.

### Luciferase reporter assay

Cells were seeded in 24-well plates and transfected with relative vectors using Lipofectamine 3000 (Invitrogen). The promoter activity was evaluated with a Dual-Luciferase Reporter Assay System (Promega, Madison, WI, USA) according to the manufacturer's instructions. Information regarding the relevant promoters and vectors is provided in Supplementary Table 6.

### DNA isolation, bisulfite conversion, and pyrosequencing assays

The genomic DNA of PCa cells was extracted through an EasyPure® Genomic DNA Kit (TransGen Biotech) and quantified using NanoDrop One C (Thermo Fisher Scientific).

Bisulfite conversion and pyrosequencing assays were performed by Shanghai Geneland Biotech Co., Ltd. (Shanghai, China). The bisulfite conversion of DNA was performed using EpiTect Bisulfite Kits (Qiagen, Hilden, Germany) according to the manufacturer's instructions.

For the pyrosequencing assay, the bisulfite-converted DNA was PCR amplified through Platinum KAPA Taq DNA Polymerase (Kapa Biosystems, Wilmington, MA, USA) according to the protocol. The primers were designed using PyroMark Assay Design Software 2.0.2 (Qiagen), and the sequences are listed in Supplementary Table 7. All reverse primers were biotinylated, and each amplified fragment contained 1 or 2 CpG sites. The biotinylated PCR products were immobilized on streptavidin-coated Sepharose beads (GE Healthcare). The pyrosequencing was performed using PyroMark Q96 ID (Qiagen) according to the manufacturer's instructions, and the percentage of CpG was calculated using PyroMark Q96 software (version 10.6) (Qiagen).

### Xenograft Studies

Xenograft assays were performed as we previously reported 26. We used 20 four-week-old male BALB/c athymic nude mice (Vital River, Beijing, China) to establish xenograft models. N-cadherin-overexpressing PC3 (PC3-sh-CDH2) cells or negative control (PC3-sh-NC) cells (1 × 10^7^) were mixed with 200 μl of phosphate-buffered saline (PBS) containing 30% Matrigel (BD Biosciences). The cells were subcutaneously inoculated into the right axilla of mice, and the mice were intraperitoneally injected with 25 mg/kg/day enzalutamide (ENZ) or the same volume of dimethyl sulfoxide (DMSO). Tumor size was measured every 5 days with a caliper, and tumor volume was calculated using the following formula: volume (mm^3^) = (length × width^2^) × 0.5. Fifty days post-inoculation, the mice were euthanized, and pathological analyses of the dissected tumors were performed. All experiments were performed in accordance with the institutional ethical guidelines of Capital Medical University.

### Histological and immunohistochemical (IHC) analyses

For pathological analyses, tumors were paraffin-embedded and sectioned at 5 μm. The sections were stained with hematoxylin and eosin (H&E) or subjected to antigen retrieval. Then, the antigen-retrieved sections were incubated with the appropriate primary and secondary antibodies. Ki-67 was used to assess tumor cell proliferation, while cleaved caspase-3 (C-Casp-3) and terminal deoxynucleotidyl transferase (TdT) dUTP nick end labeling (TUNEL) (Roche, Basel, Switzerland) were used to assess apoptosis.

Statistical analyses were also performed as we previously described 26. We randomly selected 6 fields at 400 × magnification, and the staining intensity score (1, weak; 2, moderate; and 3, strong), staining percentage score (0, ≤ 5% positive cells; 1, 6-25% positive cells; 2, 26-50% positive cells; 3, 51-75% positive cells; and 4, ≥ 76% positive cells), and staining index (SI, staining intensity score × staining percentage score) were determined as previously described^26^. The results were scored by independent observers who were blinded to each group.

### Samples from PCa patients

Sixty patients who were pathologically diagnosed with PCa (20 with GS < 7, 20 with GS = 7, and 20 with GS > 7) and underwent prostatectomy between 2016 and 2019 were recruited in accordance with the Ethics Committee of Beijing Chaoyang Hospital, affiliated with Capital Medical University. The tissues were paraffin-embedded and stored in liquid nitrogen until use.

### Statistical analysis

We performed all experiments in triplicate or more to ensure statistical significance. Comparisons of the means (continuous variables) of two groups were performed with a two-tailed Student's t-test or Mann-Whitney U test. Comparisons among ≥ 3 groups were performed with one-way or two-way analysis of variance (ANOVA), followed by post hoc Tukey's or Dunnett's multiple comparison tests. The data of categorical variables were analyzed with a chi-square (χ^2^) test. The values shown in the graphs represent the means ± standard errors of the mean (SEMs), and P < 0.05 was considered statistically significant. Pearson correlation and linear regression analyses were performed to analyze the associations between two genes. Absolute value of Pearson correlation coefficient (R) > 0.3 and P < 0.05 were considered indicative of a significant association between two genes. In the survival analysis, the Kaplan-Meier method and Cox regression model were used. The optimal cutoff value of the two groups was determined using X-tile software (https://x-tile.software.informer.com/). Statistical analyses were performed using SPSS software version 22 (IBM, Armonk, New York, USA), GraphPad Prism 7 software (GraphPad Software Inc., San Diego, CA, USA), and Microsoft Excel 2010 software.

## Results

### N-cadherin suppresses AR/NDRG1 signaling in PCa cells

**Fig 1:**
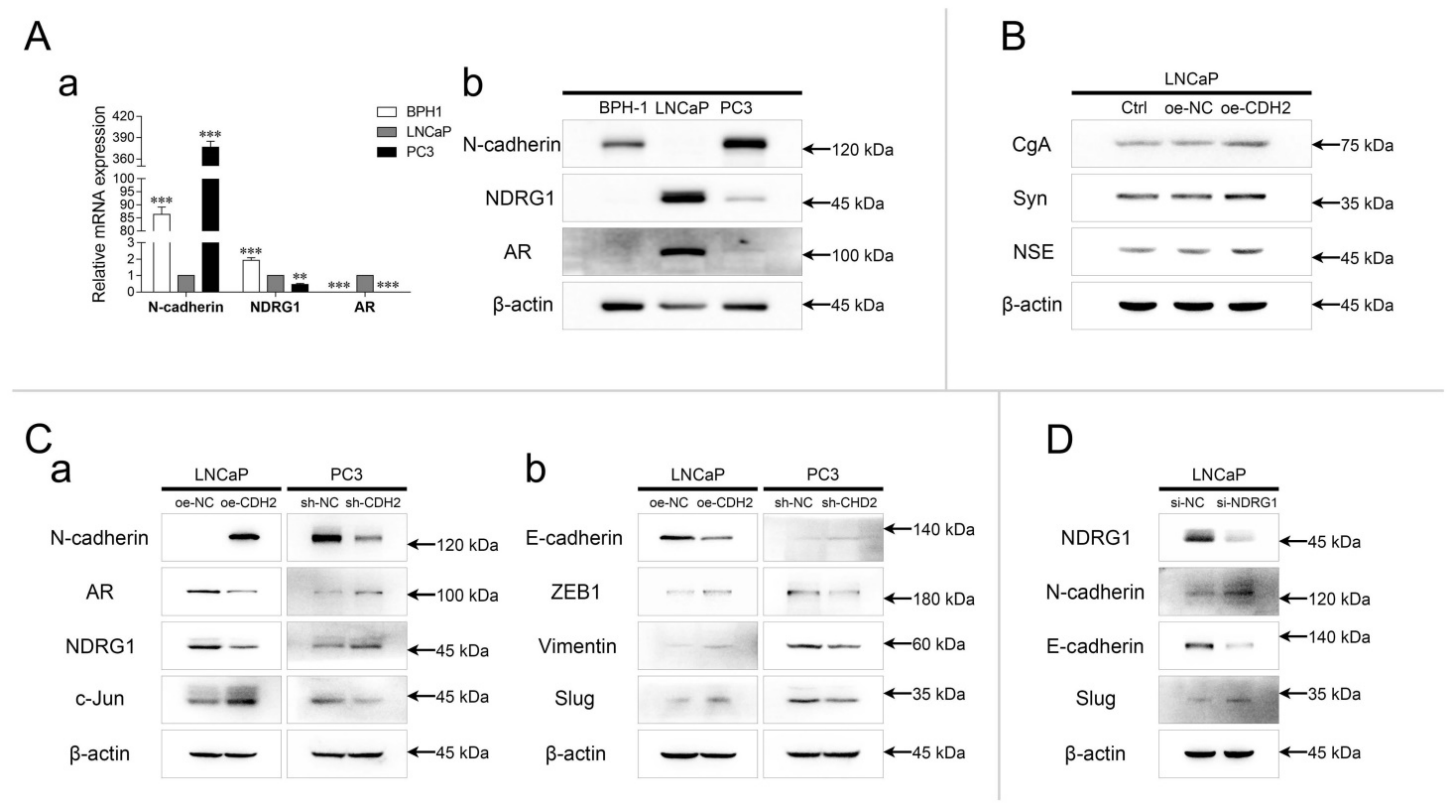
N-cadherin suppresses AR/NDRG1 signaling in PCa cells. (A) mRNA (a) and protein (b) levels of N-cadherin, NDRG1, and AR were detected in BPH-1, LNCaP, and PC3 cells (normalized to LNCaP cells) through qPCR (a) and WB (b) analyses. (B) N-cadherin was stably overexpressed in LNCaP cells via lentiviral transduction (oe-CDH2, with oe-NC as the negative control, and Ctrl as the parental LNCaP cells). WB analysis was performed with antibodies against neuroendocrine markers. (C) N-cadherin was overexpressed in LNCaP (oe-CDH2, with oe-NC as the negative control) cells and downregulated in PC3 (sh-CDH2, with sh-NC as the negative control) cells. WB was performed with AR/NDRG1 signaling-related antibodies (a) and EMT markers (b). (D) NDRG1 was transiently downregulated in LNCaP (si-NDRG1, with si-NC as the negative control) cells through siRNA transfection. WB was performed with EMT markers. Means ± SEMs are shown in the graph. *** P < 0.001.

The relative expression of N-cadherin, NDRG1, and AR was evaluated in prostate hyperplasia (BPH-1), androgen-dependent PCa (ADPC) (LNCaP), and CRPC (PC3) cell lines. The mRNA and protein levels of N-cadherin were relatively high in BPH-1 and PC3 cells, and the expression of NDRG1 and AR was significantly high in LNCaP cells (Fig. 1A). The mRNA levels of NDRG1 were relatively high, but negligible protein levels were found in BPH-1 cells. Furthermore, expression of neuroendocrine tumor markers was detected in LNCaP cells stably overexpressing N-cadherin (oe-CDH2, with oe-NC as the negative control and Ctrl as the parental LNCaP cells) via lentiviral transduction. WB analysis showed that overexpression of N-cadherin in LNCaP cells significantly promoted the expression of chromogranin A (*CgA*), synaptophysin (*Syn*), and neuron-specific enolase (*NSE*), indicating a potential relationship between EMT and the development of NEPC (Fig. 1B). In parallel with the LNCaP-oe-CDH2/NC, N-cadherin was stably downregulated in PC3 cells (sh-CDH2, with sh-NC as the negative control), and then, AR/NDRG1 signaling-related markers were detected. WB analysis showed that overexpression of N-cadherin significantly promoted the expression of c-Jun and suppressed the expression of AR and NDRG1 in LNCaP cells, while downregulation of N-cadherin in PC3 cells suppressed c-Jun expression and promoted the expression of AR and NDRG1 (Fig. 1Ca). We then assessed other EMT markers and found that N-cadherin in PCa cells promoted the expression of Zinc finger E-box binding homeobox 1 (ZEB1), Vimentin, and Slug and suppressed the expression of E-cadherin (Fig. 1Cb). To examine the regulation of N-cadherin by NDRG1 in PCa cells, NDRG1 was transiently downregulated in LNCaP cells (si-NDRG1, with si-NC as the negative control) via siRNA transfection, and EMT markers were assessed through WB analysis. Result showed that knockdown of NDRG1 in LNCaP cells promoted the expression of N-cadherin and Slug and suppressed the expression of E-cadherin (Fig. 1D).

### N-cadherin promotes PCa cell proliferation, invasion, and migration

**Fig 2:**
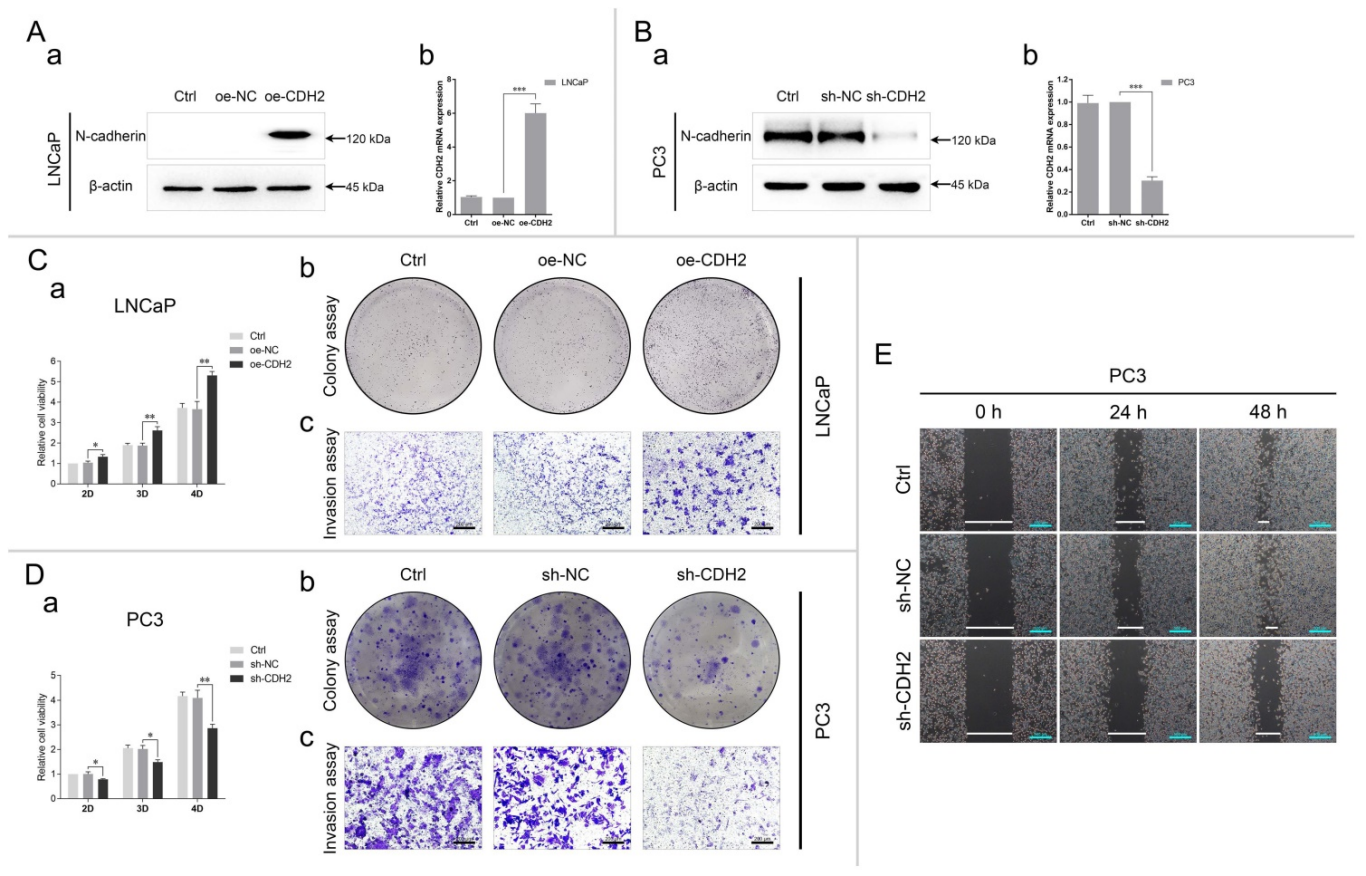
N-cadherin promotes PCa cell proliferation, invasion, and migration. (A to E) N-cadherin was stably overexpressed in LNCaP cells (oe-CDH2, oe-NC, and Ctrl) (A and C) and downregulated in PC3 cells (sh-CDH2, sh-NC, and Ctrl) (B, D, and E). (A and B) The efficiencies of gene regulation in LNCaP (A) and PC3 (B) cells were assessed by qPCR (Aa and Ba) and WB (Ab and Bb) analyses. (C to E) Cell proliferation, invasion, and migration were assessed by CCK-8 (Ca and Da), colony formation (Cb and Db), Transwell (Cc and Da), and wound healing assays (E) in LNCaP (C) and PC3 (D and E) cells with modified gene expression. Means ± SEMs are shown in the graphs. * P < 0.05, ** P < 0.01, and *** P < 0.001.

The efficiency of N-cadherin overexpression in LNCaP and knockdown in PC3 was verified through qPCR and WB analyses. Compared with the respective negative controls, the expression of N-cadherin was nearly 6 times in LNCaP-oe-CDH2 cells and 0.3 times in PC3-sh-CDH2 cells (Fig. 2A-B). Then, we assessed how N-cadherin-affected cell proliferation, invasion, and migration through CCK-8, colony formation, Transwell, and wound healing assays. The results showed that cell proliferation and invasion were promoted by overexpression of N-cadherin in LNCaP cells and cell proliferation, invasion, and migration were inhibited by knockdown of N-cadherin in PC3 cells (Fig. 2C-E). Collectively, this data confirms a prominent role for N-cadherin in the proliferation and invasion of CRPC cells.

### Knockdown of N-cadherin suppresses PCa cell invasion and migration by activating AR/NDRG1 signaling

**Fig 3:**
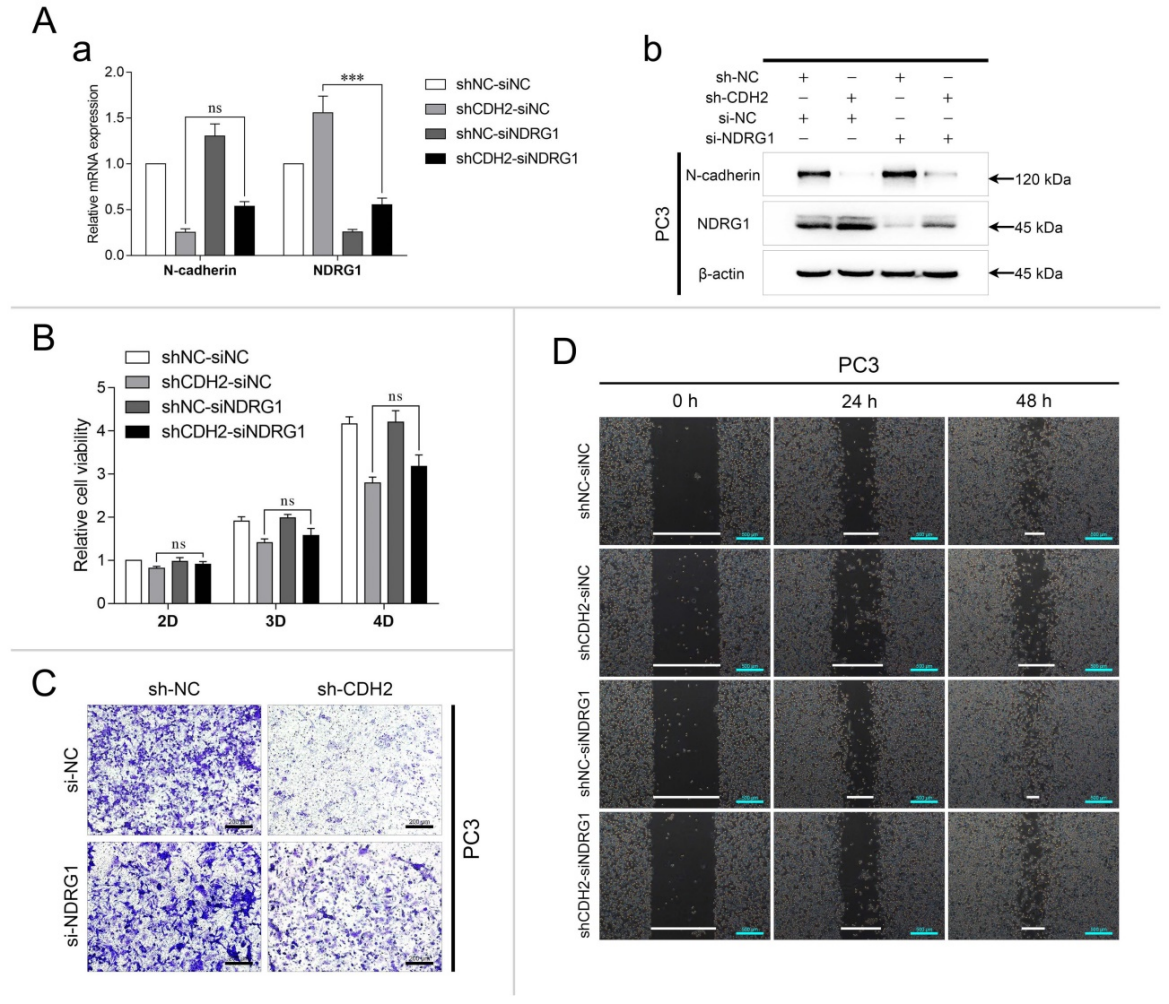
Knockdown of N-cadherin suppresses PCa cell invasion and migration by activating AR/NDRG1 signaling. (A to D) N-cadherin was stably downregulated in PC3 cells (sh-CDH2 and sh-NC), and the expression of NDRG1 was knocked down via siRNA transfection (si-NDRG1 and si-NC). mRNA levels (Aa) and protein levels (Ab) of N-cadherin and NDRG1 were assessed by qPCR and WB analyses. CCK-8 (B), Transwell (C), and wound healing (D) assays were performed in these cells. Means ± SEMs are shown in the graphs. ns P > 0.05 and *** P < 0.001.

To investigate whether N-cadherin/AR/NDRG1 signaling affects PCa progression, PC3-sh-CDH2/NC cells were transfected with an siRNA targeting NDRG1 (si-NDRG1, with si-NC as the negative control) and divided into the following four groups: shNC-siNC, shCDH2-siNC, shNC-siNDRG1, and shCDH2-siNDRG1. The experiments confirmed the efficiency of the NDRG1 siRNA in PC3 cells and showed that it was slightly influenced by N-cadherin expression (Fig. 3A). In the CCK-8 assay, NDRG1 downregulation did not affect PC3 cell proliferation, which was suppressed by knockdown of N-cadherin (Fig. 3B). However, in the Transwell and wound healing assays, NDRG1 downregulation significantly restored PC3 cell invasion and migration, which was initially suppressed by N-cadherin downregulation (Fig. 3C-D). Confirming that knockdown of N-cadherin suppresses PCa cell progression by activating AR/NDRG1 signaling.

### Downregulation of N-cadherin promotes PCa cell sensitivity to ENZ treatment

**Fig 4:**
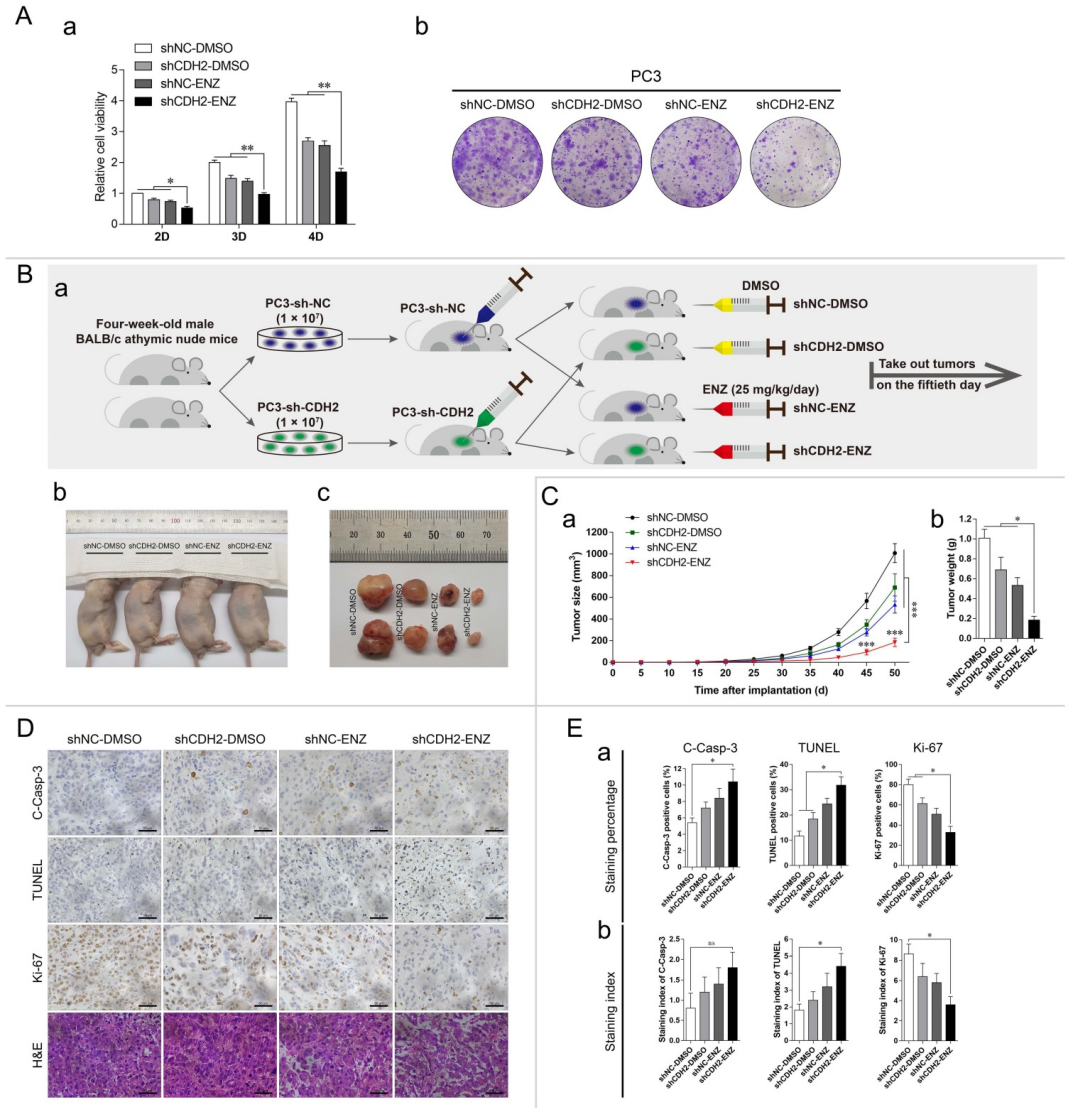
Downregulation of N-cadherin enhances PCa cell sensitivity to ENZ treatment. (A) PC3-sh-CDH2 and PC3-sh-NC cells were treated with ENZ or the same volume of DMSO. CCK-8 (a) and colony formation (b) assays were performed. (B to E) (Ba) Mice xenografted with PC3-sh-CDH2 or PC3-sh-NC cells and treated with 25 mg/kg/day ENZ or the same volume of DMSO were divided into the following four groups: shNC-DMSO, shCDH2-DMSO, shNC-ENZ, and shCDH2-ENZ. (Bb and Bc) Euthanized mice (Bb) and dissected tumors (Bc) were imaged on the fiftieth day after cell inoculation. (C) Tumor growth curves (a) and weights of the excised tumors (b) were quantitatively analyzed. (D) Representative images of IHC staining (C-Casp-3, Ki-67, and TUNEL) and H&E staining in each group. (E) Quantitative analyses of the staining percentage (a) and staining index (b) in each group. Means ± SEMs are shown in the graphs. ns P > 0.05, * P < 0.05, ** P < 0.01, and *** P < 0.001.

To determine whether N-cadherin affects the sensitivity of PCa cells to ENZ treatment, PC3-sh-CDH2/NC cells were treated with 20 μM ENZ or the same volume of DMSO. The cells were divided into the following four groups: shNC-DMSO, shCDH2-DMSO, shNC-ENZ, and shCDH2-ENZ. Cell proliferation was assessed through CCK-8 and colony formation assays. Downregulation of N-cadherin and ENZ treatment synergistically suppressed the proliferation of PC3 cells (Fig. 4A).

Based on the above results, we further performed an *in vivo* study of established xenograft mouse models. PC3-sh-CDH2/NC cells were subcutaneously inoculated into the axilla of mice, and the mice were intraperitoneally injected with 25 mg/kg/day ENZ or the same volume of DMSO. Finally, 20 animals were divided into the following four groups: shNC-DMSO, shCDH2-DMSO, shNC-ENZ, and shCDH2-ENZ (Fig. 4Ba). The mice were euthanized, and tumors were removed on the fiftieth day after xenograft model establishment (Fig. 4Bb-c). Comparison of the tumor weights and volumes between groups indicated that the shCDH2-ENZ group had the smallest tumor growth (normalized to the shNC-DMSO group: 68.5% in the shCDH2-DMSO group, 52.9% in the shNC-ENZ group, and 18.3% in the shCDH2-ENZ group) (Fig. 4C).

We then harvested the tumors and performed IHC analysis and H&E staining (Fig. 4D). The IHC staining percentage and staining index analyses demonstrated that compared with the shNC-DMSO group, the shCDH2-ENZ group exhibited higher levels of TUNEL and lower levels of Ki-67 staining in both measurements (P < 0.05) (Fig. 4E). The level of C-Casp-3 in the shCDH2-ENZ group was significantly higher than that in the shNC-DMSO group in terms of the staining percentage, but no statistically significant difference was found in the staining index (P = 0.2845) (Fig. 4E). However, the shCDH2-ENZ group exhibited an increasing trend compared with other groups. These results show that downregulation of N-cadherin synergistically affected ENZ treatment to promote tumor apoptosis and suppress tumor growth in xenograft mice. In the H&E staining, compared with the other groups, we found indistinct boundaries in cells of the shCDH2-ENZ group (Fig. 4D).

### N-cadherin is expressed at relatively high levels in advanced-stage PCa tissues

In total, 60 PCa patients were recruited from Beijing Chaoyang Hospital. The clinical characteristics are shown in Table 1. The patients were divided into two groups according to the median expression of N-cadherin and then statistically compared in terms of several important clinical data. According to the results, high expression of N-cadherin tended to be associated with lower expression of NDRG1 (P(χ^2^) < 0.001) and higher Gleason score (GS) (GS > 7) (P(χ^2^) = 0.074, P(M-W) = 0.028). Clinical T stage (P(χ^2^) = 0.282, P(M-W) = 0.064), lymph node metastasis (P(χ^2^) = 0.08), and distant metastasis (P(χ^2^) = 0.421) did not significantly differ between the groups. However, the percentages of T3 clinical stage (low:high = 20.0%:33.3%), lymph node metastasis (low:high = 16.7%:36.7%), and distant metastasis (low:high = 6.7%:16.7%) were higher in the group with high N-cadherin levels.

**Fig 5:**
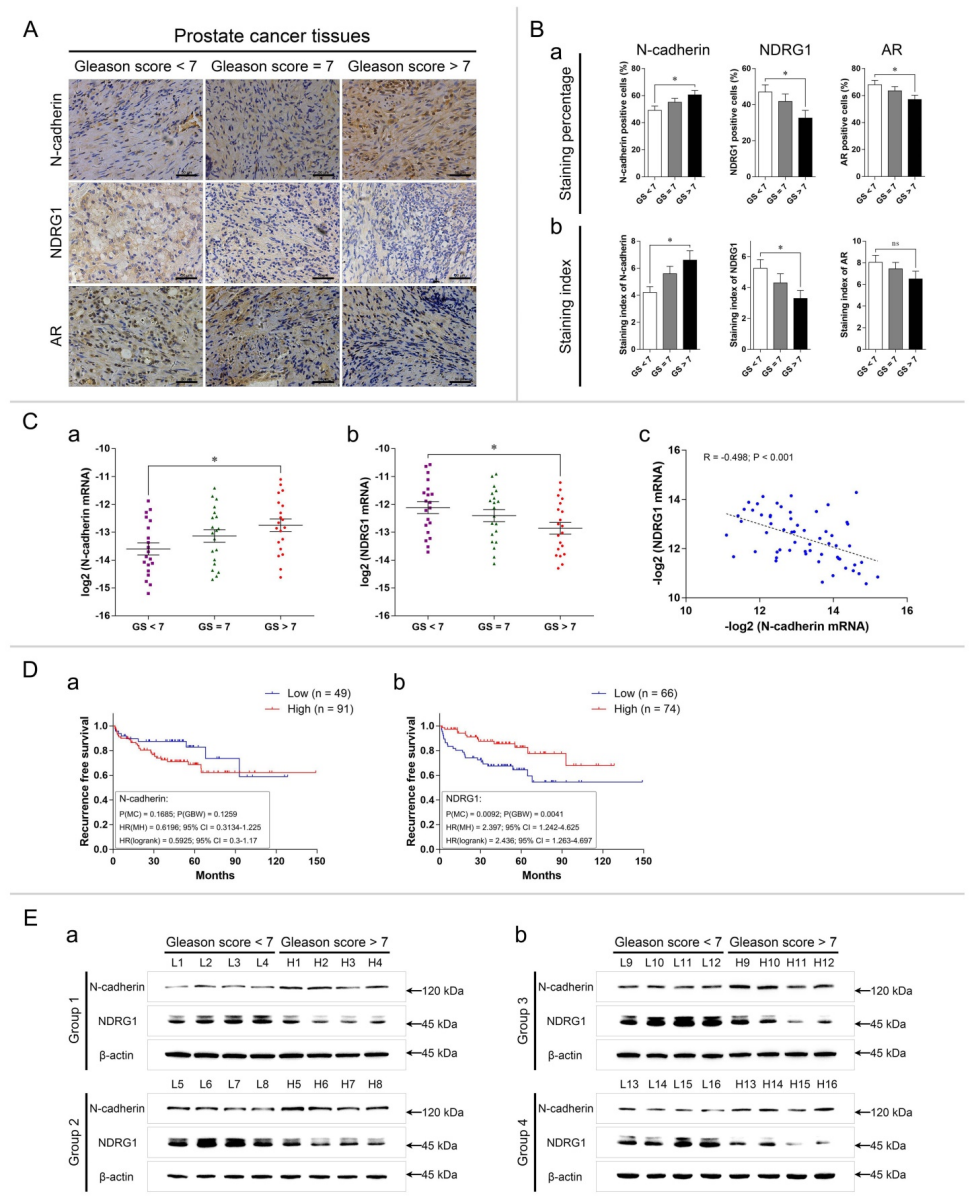
N-cadherin is expressed at relatively high levels in advanced-stage PCa tissues. (A to C) PCa tissues were divided into the following three groups: GS < 7, GS = 7, and GS > 7. (A) Representative images of IHC assays of N-cadherin, NDRG1, and AR in each group. (B) Quantitative analysis of the staining percentage (a) and staining index (b) in each group. (C) Relative log2 mRNA levels of N-cadherin (a) and NDRG1 (b) were assessed through qPCR analysis. The relationship between N-cadherin and NDRG1 -log2 mRNA levels (c) in PCa tissues was evaluated through Pearson correlation and linear regression analyses (R: correlation coefficient). (D) In the Taylor Prostate 3 database, Kaplan-Meier survival curves and Cox regression model analysis were performed to determine the association between the expression of N-cadherin (a) or NDRG1 (b) and patient RFS (cutoff value for the two groups was determined through X-tile software (Supplementary Fig. 1); P(MC): P-value from the log-rank (Mantel-Cox) test; P(GBW): P-value from the Gehan-Breslow-Wilcoxon test; HR(MH): hazard ratio (Mantel-Haenszel); HR(log-rank): hazard ratio (log-rank); 95% CI: 95% confidence interval of the HR in the Low/High groups). (E) N-cadherin and NDRG1 protein levels in PCa tissues in the GS > 7 group compared to those in the GS < 7 group through WB analysis. Means ± SEMs are shown in the graphs. ns P > 0.05 and * P < 0.05.

All PCa patients were divided into three groups based on the GSs as follows: GS < 7, GS = 7, and GS > 7. The expression of N-cadherin/AR/NDRG1-related markers was detected via IHC, qPCR, and WB analyses. The IHC staining percentage and staining index indicated that N-cadherin was upregulated while NDRG1 was downregulated in the GS > 7 group compared with those in the GS < 7 group (P < 0.05) (Fig. 5A-B). The IHC analysis further showed that compared with the GS < 7 group, the staining percentage of AR was significantly reduced in the GS > 7 group, but no significant difference was found in the staining index (P = 0.2254), even though it exhibited a trend towards decreased expression (Fig. 5B).

We then determined the relative mRNA expression of N-cadherin and NDRG1 in these groups. To satisfy a bivariate normal distribution, all values (normalized to those of 18S ribosomal RNA) were converted to log base 2 (or negative log base 2). The results showed that the mRNA expression of N-cadherin was high and that of NDRG1 was low in the CS > 7 group compared with the GS < 7 group (Fig. 5Ca-b). We also analyzed the association between N-cadherin and NDRG1 through Pearson correlation and linear regression analyses and found that N-cadherin was negatively correlated with NDRG1 in the PCa tissues (Correlation coefficient (R) = -0.498, P < 0.001) (Fig. 5Cc).

We further evaluated the effect of N-cadherin and NDRG1 expression on the prognosis of PCa patients by analyzing the Taylor Prostate 3 database. Recurrence-free survival (RFS) was assessed through Kaplan-Meier survival and Cox regression analyses. The optimal cutoff values of the two groups were determined as follows: low 49 and high 91 in N-cadherin and low 66 and high 74 in NDRG1 (determined through X-tile software) (Supplementary Fig. 1). The results showed that although the difference was not statistically significant, patients with a high expression of N-cadherin exhibited a trend towards a poor prognosis for disease recurrence and a higher hazard ratio (HR) (P(MC)_CDH2_ = 0.1685, HR(MH)_CDH2(low/high)_ = 0.6196 (0.3134-1.225)) (Fig. 5Da). Low expression of NDRG1 was associated with a significantly poor prognosis for disease recurrence and high HR (P(MC)_NDRG1_ = 0.0092, HR(MH)_NDRG1(low/high)_ = 2.397 (1.242-4.625)) (Fig. 5Db).

We selected 16 patients with GS < 7 and 16 patients with GS > 7. The patients were randomly divided into four groups, each with 4 patients with GS < 7 and 4 patients with GS > 7. WB was performed to compare the protein levels of N-cadherin and NDRG1 between the patients with GS < 7 and GS > 7 in each group. The results showed that in each group, the expression of N-cadherin was higher and that of NDRG1 was lower in the patients with GS > 7 compared with those with GS < 7 (Fig. 5E).

### Complexes of c-Jun, AR, and DNMT1 are formed on TREs in the NDRG1 promoter and suppress the transcription of NDRG1 through DNA methylation

**Fig 6:**
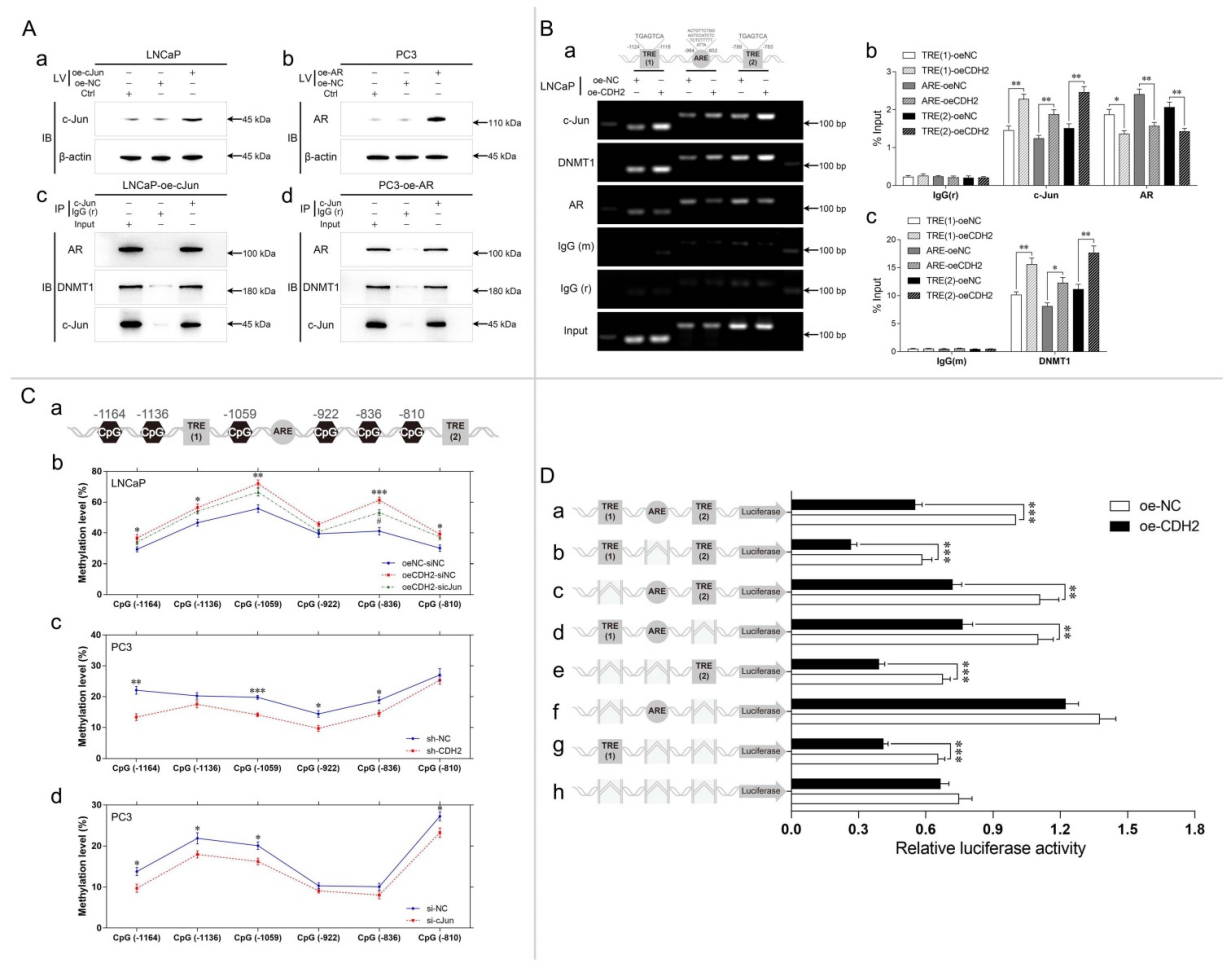
Complex formation of c-Jun, AR, and DNMT1 on the TREs in the NDRG1 promoter suppresses the transcription of NDRG1 through DNA methylation. (A) c-Jun was overexpressed in LNCaP cells (LNCaP-oe-cJun) (a and c), and AR was overexpressed in PC3 (PC3-oe-AR) (b and d) cells. The gene regulation efficiency was determined through WB analysis (a and b). A Co-IP assay was performed with LNCaP-oe-cJun (c) and PC3-oe-AR (d) cells through immunoprecipitation with anti-cJun or same-host-sourced anti-IgG antibody and immunoblotting with anti-AR, anti-DNMT1, and anti-cJun antibodies (IV: lentiviral transduction. IP: immunoprecipitation; IB: immunoblotting). (B) ChIP assays were performed with LNCaP-oe-NC and LNCaP-oe-CDH2 cells through immunoprecipitation with anti-cJun, anti-DNMT1, anti-AR, anti-IgG (mouse, m), and anti-IgG (rabbit, r) antibodies. The TRE (1), ARE, and TRE (2) of NDRG1 were assayed by PCR (a) and qPCR (b and c) analysis. (C) (Ca) The positions of CpG sites near TRE (1), ARE, or TRE (2) in the NDRG1 promoter are shown as schematic diagram. (Cb to Cd) LNCaP-oe-CDH2 cells with transient downregulation of c-Jun expression were divided into three groups with negative controls (oeCDH2-sicJun, oeCDH2-siNC, and oeNC-siNC) (* statistical significance of oeNC-siNC vs oeCDH2-siNC, # statistical significance of oeCDH2-siNC vs oeCDH2-sicJun) (b); PC3 cells with stable downregulation of N-cadherin expression (sh-CDH2 and sh-NC) (c) or transient downregulation of c-Jun expression (si-cJun and si-NC) (d) were established. DNA methylation rates of the six CpG sites in the generated PCa cell lines were assessed through pyrosequencing assay. (D) Carrier vectors containing specific promoter regions of NDRG1 (wild-type or deficient TRE and/or ARE) (a, wild-type; b, ARE deletion (ΔARE); c, ΔTRE (1); d, ΔTRE (2); e, ΔTRE (1) and ΔARE; f, ΔTRE (1) and ΔTRE (2); g, ΔARE and ΔTRE (2); h, ΔTRE (1), ΔARE, and ΔTRE (2)) were transfected at equal volumes into LNCaP-oe-NC and LNCaP-oe-CDH2 cells. A dual-luciferase reporter assay was performed to estimate the activity of NDRG1 promoter. Means ± SEMs are shown in the graphs. * and # P < 0.05, ** P < 0.01, and *** P < 0.001.

To achieve high expression of both c-Jun and AR in PCa cells, c-Jun was overexpressed in LNCaP (LNCaP-oe-cJun) cells, and AR was overexpressed in PC3 (PC3-oe-AR) cells through lentiviral transduction. Gene regulation was assessed through WB analysis (Fig. 6Aa-b). A Co-IP assay was performed in both cell lines through immunoprecipitation with anti-cJun or same-host-sourced anti-IgG antibodies, and immunoblotting was performed with anti-AR, anti-DNMT1, and anti-cJun antibodies. The results showed that c-Jun immunoprecipitated with AR and DNMT1, demonstrating complex formation of c-Jun, AR, and DNMT1 (Fig. 6Ac-d).

Two adjacent TREs (TRE (1) and TRE (2)) and an ARE were found in the promoter of NDRG1 (Fig. 6Ba). To evaluate whether N-cadherin-induced c-Jun and AR transcriptional activity in the promoter of NDRG1, a ChIP assay was performed with LNCaP-oe-CDH2/NC cells. The cross-linked lysates were immunoprecipitated with anti-cJun, anti-DNMT1, anti-AR, and same-host-sourced IgG (mouse or rabbit) antibodies. Then, TRE (1), ARE, and TRE (2) in the NDRG1 promoter were detected through PCR and qPCR. The results showed that overexpression of N-cadherin significantly promoted c-Jun and DNMT1 binding to the TREs but suppressed AR binding to the ARE of the NDRG1 promoter region (Fig. 6B). Although we found that c-Jun also binds to the ARE, the distances between the TREs and ARE sequences were too close (less than 200 bp); thus, there may have been an error during DNA shearing by sonication of the lysates.

Then, we investigated whether the expression of c-Jun affected DNA methylation near TREs of NDRG1 promoter. DNA methylation rates were quantitatively assessed by pyrosequencing analysis across an extended region of 356 bp spanning six CpG sites (CpG (-1164), CpG (-1136), CpG (-1059), CpG (-922), CpG (-836), and CpG (-810)), which are close to the TRE sequences in the NDRG1 promoter (Fig. 6Ca). We generated LNCaP-oe-CDH2 cells with transient downregulation of c-Jun expression (oeCDH2-sicJun, oeCDH2-siNC, and oeNC-siNC) and PC3 cells with stable downregulation of N-cadherin (sh-CDH2 and sh-NC) or transient downregulation of c-Jun expression (si-cJun and si-NC). Overexpression of N-cadherin in LNCaP cells significantly increased methylation in 5 CpG sites (CpG (-1164), CpG (-1136), CpG (-1059), CpG (-836), and CpG (-810)) (P < 0.05), while concomitant downregulation of c-Jun markedly decreased DNA methylation in the CpG sites (statistically significant in CpG (-836)) (Fig. 6Cb). In PC3 cells, downregulation of N-cadherin significantly decreased DNA methylation in 4 CpG sites (CpG (-1164), CpG (-1059), CpG (-922), and CpG (-836)) (P < 0.05), and downregulation of c-Jun also decreased DNA methylation in 4 CpG sites (CpG (-1164), CpG (-1136), CpG (-1059), and CpG (-810)) (Fig. 6Cb). This data confirms that N-cadherin promotes c-Jun-induced DNA methylation in the promoter of NDRG1.

**Fig 7:**
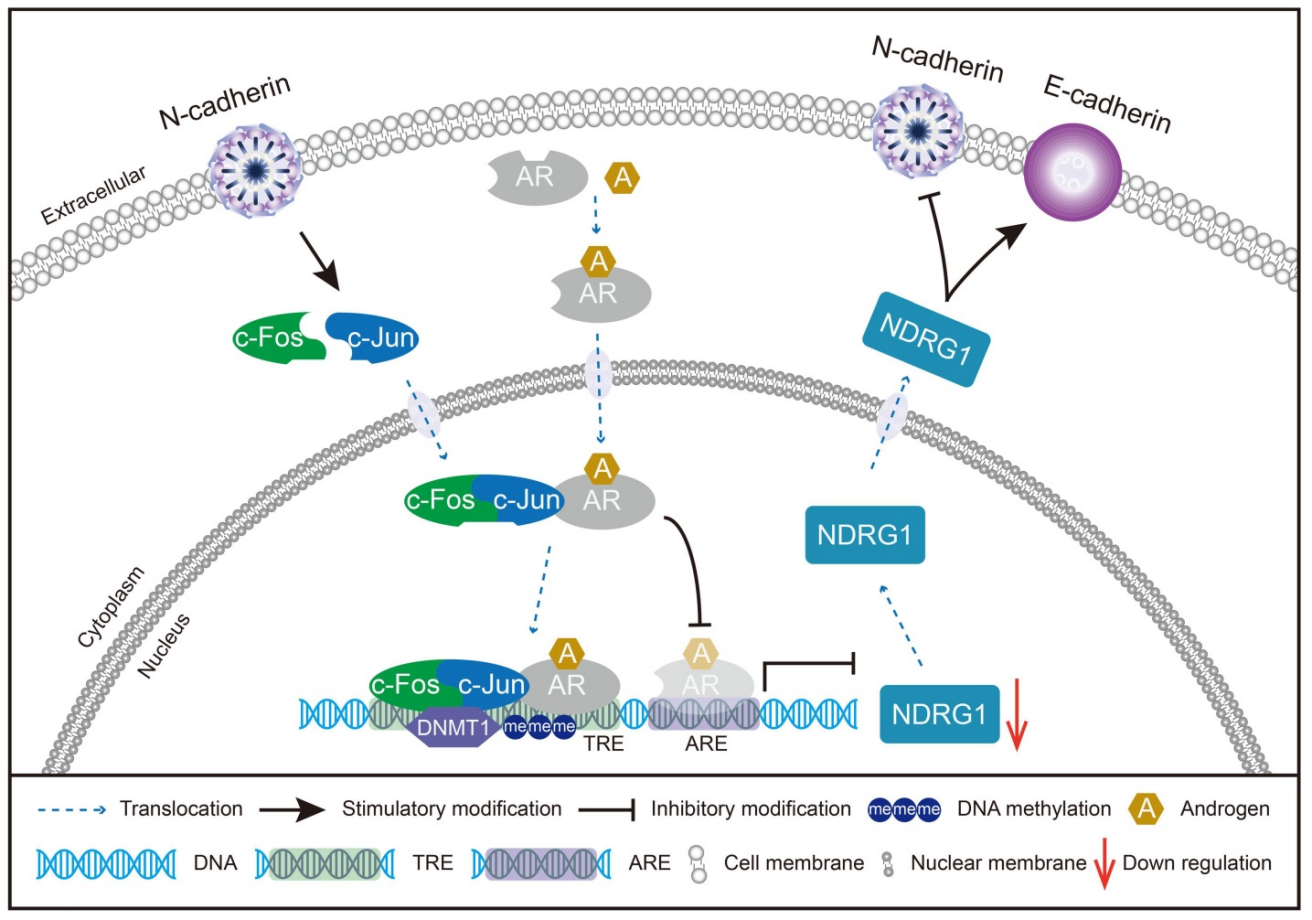
Schematic model of the potential mechanisms involved in CRPC progression through the N-cadherin/c-Jun/NDRG1 axis. c-Jun is promoted by N-cadherin to induce heterodimerization with c-Fos to form AP-1. AP-1 forms a complex with AR and binds to TRE rather than ARE on the promoter of NDRG1. Then, AP-1 interacts with DNMT1 and further promotes DNA methylation to suppress the transcription of NDRG1. Furthermore, the decrease in NDRG1 expression reduces its role as an EMT marker and promotes the expression of N-cadherin to form a vicious cycle, ultimately leading to CRPC progression.

Whether N-cadherin suppression of NDRG1 expression is caused by TREs is unclear and we cannot exclude other mechanisms independent of TREs leading to DNA methylation caused by N-cadherin or c-Jun. Therefore, a dual-luciferase reporter assay was performed by transfecting LNCaP-oe-NC/CDH2 cells with vectors carrying specific deletions of the TREs and/or ARE in the NDRG1 promoter. The results showed that overexpression of N-cadherin in LNCaP cells did not significantly repress the transcription of the NDRG1 promoter when both TRE (1) and TRE (2) were deleted (Fig. 6Df and Dh), indicating that the process is dependent on c-Jun-regulated TRE sequences. ARE was not affected by N-cadherin but has a role in transcriptional activation because luciferase activity was increased when it was added, and overexpression of N-cadherin could not suppress such activation (f versus (vs) h in Fig. 6D). However, further addition of the TRE sequences significantly reduced the ARE-induced luciferase activity (oe-CDH2 of a, c, and d vs f in Fig. 6D). Considering the complex formation of c-Jun and AR, there may be a potential mechanism by which the N-cadherin-induced cJun-AR complex may preferentially bind to TREs, and this dominant interaction competitively inhibits AR binding to the ARE, ultimately promoting AR-independent progression of PCa (Fig. 7).

## Discussion

In this study, we elucidated a novel mechanism of the effect of the c-Jun/NDRG1/N-cadherin axis on the progression of PCa. This study provides the first evidence of complex formation between c-Jun, AR, and DNMT1, which is promoted by N-cadherin. Furthermore, we showed that this complex epigenetically suppresses AR/NDRG1 signaling to promote the progression of CRPC.

N-cadherin has been reported to promote PCa cell invasion and migration via several pathways, such as through ErbB signaling 42, 43. It was also found to be upregulated in the advanced stage of PCa and was associated with a poor prognosis 10, 44, 63. However, few studies focused on demonstrating a molecular mechanism for how N-cadherin mediated the transformation of ADPC to CRPC.

To solve this problem, we used the ADPC cell line LNCaP and the CRPC cell line PC3. PC3 was previously reported as an AR-negative cell line 64-66. However, we couldn't exclude low expression of AR in PC3 cells through comparison with LNCaP cells. Several studies including present results confirmed that PC3 cells express detectable levels of AR 26, 67-72 and that PC3 also demonstrates sensitivity to high concentrations of ENZ 73. It has also been reported that low expression of AR in PC3 is caused by DNA methylation of the AR promoter, which is a major mechanism underlying resistance to ADT 64, 71, 74.

We found that N-cadherin significantly promoted the expression of other EMT-related markers (Slug, Vimentin, and ZEB1) and neuroendocrine markers (CgA, Syn, and NSE) in PCa cells, suggesting that N-cadherin may promote PCa cell proliferation, invasion, and migration by regulating EMT and NEPC transformation.

According to accepted theories that N-cadherin functional activation and AR repression by ADT are major causes of CRPC progression 7-19, there may be significant molecular mechanisms that induce an inverse correlation between N-cadherin and AR. In the present study, we found knockdown of NDRG1 in LNCaP cells could significantly promote the expression of N-cadherin and Slug. Furthermore, our previous study elucidated a mechanism by which AR epigenetically activates NDRG1 via histone methylation to suppress the expression of N-cadherin 26, suggesting mechanistic interplay between these two critical proteins.

However, in the course of further study, we found that N-cadherin, a downstream effector, could reversibly inhibit the expression of AR and NDRG1 in PCa cells, suggesting that N-cadherin is not only a downstream effector but that an interplay mechanism may exist between N-cadherin and NDRG1. We further demonstrated that N-cadherin promotes PCa invasion and migration through suppressing AR/NDRG1 signaling and that other mechanisms may participate in N-cadherin-induced cell proliferation, which is consistent with the notion that NDRG1 does not affect PCa cell proliferation 26, 30, 31.

The specific mechanism by which N-cadherin affects AR/NDRG1 signaling is unclear. We found that N-cadherin significantly promoted the expression of c-Jun, which is a transcription factor that binds to the TRE to regulate several target genes 46. A growing body of studies recently suggested that c-Jun negatively regulates AR and AR transcriptional activity in PCa 49-58.

The regulatory effect of c-Jun on transcription repression is achieved through DNA methylation 59-61, which is the most stable and best characterized epigenetic modification 75. DNA methylation occurs at the cytosine of CpG dinucleotides and is catalyzed by DNMT family members, such as DNMT1, DNMT3a, and DNMT3b, to silence gene transcription 76.

We initially hypothesized that N-cadherin induces c-Jun to bind to the promoter of AR and suppress AR expression through DNA methylation. However, only one TRE sequence (position -3193 to -3187) was found in the promoter region of AR (position -6000 to -1), and no CpG site was found at approximately 150 bp of TRE, suggesting that c-Jun is unlikely to induce DNA methylation at the AR promoter by binding to the TRE.

We then considered another mechanism by which N-cadherin induces c-Jun to bind to the TREs of NDRG1 and competitively regulate its transcriptional activity with AR. In the NDRG1 promoter region (position -2000 to -1), we found two TREs (TRE (1) in position -1124 to -1118 and TRE (2) in position -789 to -783) located near the ARE (position -984 to -952). c-Jun has been reported to form a complex with AR 47, 58, 77 and DNMT1 61. However, c-Jun and AR were negatively regulated, and previous studies simply used LNCaP cells to perform Co-IP assays. To avoid false positive results and further confirm the interaction among c-Jun, AR, and DNMT1, we overexpressed c-Jun in LNCaP (LNCaP-oe-cJun) cells and overexpressed AR in PC3 (PC3-oe-AR) cells. Collectively, the Co-IP results confirmed complex formation between c-Jun, AR, and DNMT1 in PCa cells.

The influence of the AR-ARE interaction on AP-1 is controversial because c-Jun has been reported to either competitively inhibit 52 or have no effect 53 on AR binding to the ARE. Our results suggested that N-cadherin promotes c-Jun binding to the TREs and suppresses AR binding to the ARE on the promoter region of NDRG1. Complex formation of c-Jun, AR, and DNMT1 suggests that c-Jun competitively represses AR transcriptional activity through combine with AR and interfering the AR-ARE binding efficiency. Present study further confirmed that N-cadherin and c-jun significantly recruits DNMT1 to the TREs and induces DNA methylation in the promoter of NDRG1. These findings describe the potential mechanism by which N-cadherin induces the cJun-AR complex to bind to TREs and suppresses the transcription of NDRG1.

In our previous study, the sensitivity of CRPC cells to ENZ treatment was restored by epigenetically stimulating AR/NDRG1 signaling 26. Therefore, we assessed whether downregulation of N-cadherin in PC3 cells could restore sensitivity to ENZ treatment. *In vitro* and *in vivo* assays both confirmed the synergistic antiproliferative effects of N-cadherin downregulation and ENZ treatment. The expression of apoptosis marker (TUNEL) was also significantly upregulated under both treatments, suggesting that N-cadherin knockdown may induced the transition of CRPC to ADPC in PC3 cells.

In PCa patients, GS ≥ 7 has been defined as an important predictor of PCa aggressiveness 78, 79. According to this surrogate marker, the PCa tissues were then divided into the following three groups: GS < 7, GS = 7, and GS > 7. We found PCa patients with higher expression of N-cadherin tended to be associated with lower expression of NDRG1 (P < 0.05). The Taylor Prostate 3 database analysis identified poor RFS and a higher HR in the groups with higher N-cadherin and lower NDRG1 expression. All conclusions with PCa patients were consistent with our previous results that N-cadherin promotes PCa progression by suppressing AR/NDRG1 signaling.

Several limitations in this study warrant further research. First, N-cadherin was confirmed to suppress AR expression, but the specific mechanism underlying the relationship was undetermined, which may be the reason that functional amplification of c-Jun competitively repressed AR transcriptional activity and further led to degeneration of AR. Additionally, the specific mechanism by which N-cadherin promoted c-Jun is unclear in this study. Finally, the N-cadherin-induced promotion of PCa cell proliferation is independent of NDRG1 expression, and other molecular mechanisms may participate in this process.

## Conclusions

In this study, we elucidated a mechanism underlying CRPC progression through the N-cadherin/c-Jun/NDRG1 axis. N-cadherin is highly expressed in malignant PCa tissues and epigenetically suppresses AR/NDRG1 signaling through c-Jun. c-Jun forms a complex with AR and DNMT1 on the TREs of the NDRG1 promoter and not only promotes DNA methylation through DNMT1 but also competitively suppresses AR-induced transcriptional activity on the ARE in the NDRG1 promoter. Preventing this vicious cycle by repressing the expression of N-cadherin may shed light on ways to overcome CRPC progression and reverse CRPC to ADPC.

## Supplementary Material

Supplementary figure and tables.

## Figures and Tables

**Table 1:** Correlations between N-cadherin expression and PCa patients' clinical characteristics.

Clinicopathological parameters	Total (n = 60) (%)	N-cadherin expression^a^		P-value S-T^b^/M-W^c^/χ^2d^ test
		Low (%)	High (%)	
**Age**				
Median (IQR)	66 (61-69.75)	66 (59-68.25)	66.5 (62.75-71)	0.191^b^
Range (Min, Max)	55-76	55-76	57-75	
< 65	24 (40.0%)	13 (43.3%)	11 (36.7%)	0.598^d^
≥ 65	36 (60.0%)	17 (56.7%)	19 (63.3%)	
**Log2 NDRG1 expression^a^**				
Median (IQR)	-12.4777 (-13.3336--11.6622)	-11.8416 (-12.5765--11.3095)	-13.069 (-13.5803--12.1849)	0.000^b^*
Range (Min, Max)	-14.2934--10.5782	-14.2934--10.5782	-14.1577--11.507	
Low	30 (50.0%)	8 (26.7%)	22 (73.3%)	0.000^d^
High	30 (50.0%)	22 (73.3%)	8 (26.7%)	
**Total PSA (t-PSA)**				
Median (IQR)	17.275 (8.5725-42.4225)	19.76 (8.3675-42.3)	16.285 (8.3325-43.8875)	0.918^c^
Range (Min, Max)	0.56-85.35	1.05-64.24	0.56-85.35	
< 4 ng/ml	5 (8.3%)	3 (10.0%)	2 (6.7%)	0.517^d^ 0.404^c^
4-10 ng/ml	15 (25.0%)	5 (16.7%)	10 (33.3%)	
10-20 ng/ml	13 (21.7%)	7 (23.3%)	6 (20.0%)	
> 20 ng/ml	27 (45.0%)	15 (50.0%)	12 (40.0%)	
**Gleason Score (GS)**				
< 7	20 (33.3%)	14 (46.7%)	6 (20.0%)	0.074^d^ 0.028^c^*
7	20 (33.3%)	9 (30.0%)	11 (36.7%)	
> 7	20 (33.3%)	7 (23.3%)	13 (43.3%)	
**Clinical T-stage**				
T2a	7 (11.7%)	5 (16.7%)	2 (6.7%)	0.282^d^ 0.064^c^
T2b	14 (23.3%)	9 (30.0%)	5 (16.7%)	
T2c	23 (38.3%)	10 (33.3%)	13 (43.3%)	
T3a or T3b	16 (26.7%)	6 (20.0%)	10 (33.3%)	
**Lymph node metastasis**				
N0	44 (73.3%)	25 (83.3%)	19 (63.3%)	0.080^d^
N1	16 (26.7%)	5 (16.7%)	11 (36.7%)	
**Distant metastasis**				
M0 or Mx	53 (88.3%)	28 (93.3%)	25 (83.3%)	0.421^d^
M1	7 (11.7%)	2 (6.7%)	5 (16.7%)	
**TNM stage**				
I-II	35 (58.3%)	19 (63.3%)	16 (53.3%)	0.432^d^
III-IV	25 (41.7%)	11 (36.7%)	14 (46.7%)	

^*^ P < 0.05 ^a^ Median mRNA expression of N-cadherin as a cutoff. ^b^ P-value (2-sided) of Student's t-test. ^c^ P-value (2-sided) of the Mann-Whitney U test. ^d^ P-value (2-sided) of Pearson chi-square test or continuity correction for the chi-square test.

## Abbreviations

AR: androgen receptor
PCa: prostate cancer
ADT: androgen deprivation therapy
CRPC: castration-resistant prostate cancer
CCK-8: Cell Counting Kit-8
WB: western blot
IHC: immunohistochemistry
qPCR: quantitative real-time PCR
Co-IP: co-immunoprecipitation
ChIP: chromatin immunoprecipitation
NDRG1: N-myc downstream regulated gene
EMT: epithelial-to-mesenchymal transition
ENZ: enzalutamide
GS: Gleason score
TRE: 12-O-tetradecanoyl phorbol-13-acetate (TPA) response element
ADPC: androgen-dependent prostate cancer
NEPC: neuroendocrine prostate cancer
ARE: androgen response element
DHT: dihydrotestosterone
R1881: methyltrienolone
CDH2: N-cadherin, also called Cadherin-2
bZIP: basic leucine zipper
AP-1: activator protein-1
DNMT1: DNA methyltransferase-1
FBS: fetal bovine serum
AA: antibiotic-antimycotic
shRNA: short hairpin RNA
h: hour/hours
siRNA: small interfering RNA
cDNA: complementary DNA
RIPA: radioimmunoprecipitation assay
SDS-PAGE: SDS-polyacrylamide gel electrophoresis
PVDF: polyvinylidene difluoride
PBS: phosphate-buffered saline
DMSO: dimethyl sulfoxide
H&E: hematoxylin and eosin
TUNEL: terminal deoxynucleotidyl transferase (TdT) dUTP nick end labeling
SI: staining index
ANOVA: analysis of variance
SEMs: standard errors of the mean
R: pearson correlation coefficient
CgA: chromogranin A
Syn: synaptophysin
NSE: neuron-specific enolase
vs: Versus
DNMT: DNA methyltransferase
ZEB1: zinc finger E-box binding homeobox 1
C-Casp-3: cleaved caspase-3
RFS: recurrence-free survival
MC: Mantel-Cox test
GBW: Gehan-Breslow-Wilcoxon test
MH: Mantel-Haenszel
HR: hazard ratio
CI: confidence interval
IP: immunoprecipitation
IB: immunoblotting
m: mouse
r: rabbit
Δ: deletion
χ: Pearson chi-square test
M-W: Mann-Whitney U test
S-T: Student's t-test
IQR: interquartile range

## References

[1] Siegel RL, Miller KD, Jemal A (2019). Cancer statistics, 2019. CA: a cancer journal for clinicians.

[2] Attard G, Parker C, Eeles RA, Schröder F, Tomlins SA, Tannock I (2016). Prostate cancer. Lancet (London, England).

[3] Jacob S, Nayak S, Fernandes G, Barai RS, Menon S, Chaudhari UK (2014). Androgen receptor as a regulator of ZEB2 expression and its implications in epithelial-to-mesenchymal transition in prostate cancer. Endocrine-related cancer.

[4] Yuan X, Cai C, Chen S, Chen S, Yu Z, Balk SP (2014). Androgen receptor functions in castration-resistant prostate cancer and mechanisms of resistance to new agents targeting the androgen axis. Oncogene.

[5] Wen S, Niu Y, Lee SO, Chang C (2014). Androgen receptor (AR) positive vs negative roles in prostate cancer cell deaths including apoptosis, anoikis, entosis, necrosis and autophagic cell death. Cancer treatment reviews.

[6] Niu Y, Chang TM, Yeh S, Ma WL, Wang YZ, Chang C (2010). Differential androgen receptor signals in different cells explain why androgen-deprivation therapy of prostate cancer fails. Oncogene.

[7] Niu Y, Altuwaijri S, Yeh S, Lai KP, Yu S, Chuang KH (2008). Targeting the stromal androgen receptor in primary prostate tumors at earlier stages. Proceedings of the National Academy of Sciences of the United States of America.

[8] Niu Y, Altuwaijri S, Lai KP, Wu CT, Ricke WA, Messing EM (2008). Androgen receptor is a tumor suppressor and proliferator in prostate cancer. Proceedings of the National Academy of Sciences of the United States of America.

[9] Jia L, Wu D, Wang Y, You W, Wang Z, Xiao L (2018). Orphan nuclear receptor TLX contributes to androgen insensitivity in castration-resistant prostate cancer via its repression of androgen receptor transcription. Oncogene.

[10] Jennbacken K, Tesan T, Wang W, Gustavsson H, Damber JE, Welen K (2010). N-cadherin increases after androgen deprivation and is associated with metastasis in prostate cancer. Endocrine-related cancer.

[11] Mendiratta P, Mostaghel E, Guinney J, Tewari AK, Porrello A, Barry WT (2009). Genomic strategy for targeting therapy in castration-resistant prostate cancer. Journal of clinical oncology: official journal of the American Society of Clinical Oncology.

[12] Sun Y, Wang BE, Leong KG, Yue P, Li L, Jhunjhunwala S (2012). Androgen deprivation causes epithelial-mesenchymal transition in the prostate: implications for androgen-deprivation therapy. Cancer research.

[13] Chen J, Li L, Yang Z, Luo J, Yeh S, Chang C (2017). Androgen-deprivation therapy with enzalutamide enhances prostate cancer metastasis via decreasing the EPHB6 suppressor expression. Cancer letters.

[14] Qin J, Lee HJ, Wu SP, Lin SC, Lanz RB, Creighton CJ (2014). Androgen deprivation-induced NCoA2 promotes metastatic and castration-resistant prostate cancer. The Journal of clinical investigation.

[15] Chen WY, Tsai YC, Siu MK, Yeh HL, Chen CL, Yin JJ (2017). Inhibition of the androgen receptor induces a novel tumor promoter, ZBTB46, for prostate cancer metastasis. Oncogene.

[16] Tsai YC, Chen WY, Abou-Kheir W, Zeng T, Yin JJ, Bahmad H (2018). Androgen deprivation therapy-induced epithelial-mesenchymal transition of prostate cancer through downregulating SPDEF and activating CCL2. Biochim Biophys Acta Mol Basis Dis.

[17] Cai Q, Chen Y, Zhang D, Pan J, Xie Z, Ma S (2020). Loss of epithelial AR increase castration resistant stem-like prostate cancer cells and promotes cancer metastasis via TGF-β1/EMT pathway. Translational andrology and urology.

[18] Miao L, Yang L, Li R, Rodrigues DN, Crespo M, Hsieh JT (2017). Disrupting Androgen Receptor Signaling Induces Snail-Mediated Epithelial-Mesenchymal Plasticity in Prostate Cancer. Cancer research.

[19] Huo C, Kao YH, Chuu CP (2015). Androgen receptor inhibits epithelial-mesenchymal transition, migration, and invasion of PC-3 prostate cancer cells. Cancer letters.

[20] Komiya A, Yasuda K, Watanabe A, Fujiuchi Y, Tsuzuki T, Fuse H (2013). The prognostic significance of loss of the androgen receptor and neuroendocrine differentiation in prostate biopsy specimens among castration-resistant prostate cancer patients. Mol Clin Oncol.

[21] Beltran H, Rickman DS, Park K, Chae SS, Sboner A, MacDonald TY (2011). Molecular characterization of neuroendocrine prostate cancer and identification of new drug targets. Cancer Discov.

[22] Beltran H, Prandi D, Mosquera JM, Benelli M, Puca L, Cyrta J (2016). Divergent clonal evolution of castration-resistant neuroendocrine prostate cancer. Nat Med.

[23] Chen WY, Zeng T, Wen YC, Yeh HL, Jiang KC, Chen WH (2019). Androgen deprivation-induced ZBTB46-PTGS1 signaling promotes neuroendocrine differentiation of prostate cancer. Cancer letters.

[24] Zhang Y, Zheng D, Zhou T, Song H, Hulsurkar M, Su N (2018). Androgen deprivation promotes neuroendocrine differentiation and angiogenesis through CREB-EZH2-TSP1 pathway in prostate cancers. Nature communications.

[25] Pertschuk LP, Schaeffer H, Feldman JG, Macchia RJ, Kim YD, Eisenberg K (1995). Immunostaining for prostate cancer androgen receptor in paraffin identifies a subset of men with a poor prognosis. Lab Invest.

[26] Quan Y, Cui Y, Wahafu W, Liu Y, Ping H, Zhang X (2020). MLL5α activates AR/NDRG1 signaling to suppress prostate cancer progression. American journal of cancer research.

[27] Bae DH, Jansson PJ, Huang ML, Kovacevic Z, Kalinowski D, Lee CS (2013). The role of NDRG1 in the pathology and potential treatment of human cancers. J Clin Pathol.

[28] Sun J, Zhang D, Bae DH, Sahni S, Jansson P, Zheng Y (2013). Metastasis suppressor, NDRG1, mediates its activity through signaling pathways and molecular motors. Carcinogenesis.

[29] Song Y, Oda Y, Hori M, Kuroiwa K, Ono M, Hosoi F (2010). N-myc downstream regulated gene-1/Cap43 may play an important role in malignant progression of prostate cancer, in its close association with E-cadherin. Human pathology.

[30] Bandyopadhyay S, Pai SK, Gross SC, Hirota S, Hosobe S, Miura K (2003). The Drg-1 gene suppresses tumor metastasis in prostate cancer. Cancer research.

[31] Sharma A, Mendonca J, Ying J, Kim HS, Verdone JE, Zarif JC (2017). The prostate metastasis suppressor gene NDRG1 differentially regulates cell motility and invasion. Molecular oncology.

[32] Kim KH, Dobi A, Shaheduzzaman S, Gao CL, Masuda K, Li H (2007). Characterization of the androgen receptor in a benign prostate tissue-derived human prostate epithelial cell line: RC-165N/human telomerase reverse transcriptase. Prostate Cancer Prostatic Dis.

[33] Masuda K, Werner T, Maheshwari S, Frisch M, Oh S, Petrovics G (2005). Androgen receptor binding sites identified by a GREF_GATA model. J Mol Biol.

[34] Hu ZY, Xie WB, Yang F, Xiao LW, Wang XY, Chen SY (2015). NDRG1 attenuates epithelial-mesenchymal transition of nasopharyngeal cancer cells via blocking Smad2 signaling. Biochimica et biophysica acta.

[35] Lee JC, Chung LC, Chen YJ, Feng TH, Juang HH (2014). N-myc downstream-regulated gene 1 downregulates cell proliferation, invasiveness, and tumorigenesis in human oral squamous cell carcinoma. Cancer letters.

[36] Chiang KC, Yeh CN, Chung LC, Feng TH, Sun CC, Chen MF (2015). WNT-1 inducible signaling pathway protein-1 enhances growth and tumorigenesis in human breast cancer. Scientific reports.

[37] Lingadahalli S, Jadhao S, Sung YY, Chen M, Hu L, Chen X (2018). Novel lncRNA LINC00844 Regulates Prostate Cancer Cell Migration and Invasion through AR Signaling. Molecular cancer research: MCR.

[38] Ferreira LB, Palumbo A, de Mello KD, Sternberg C, Caetano MS, de Oliveira FL (2012). PCA3 noncoding RNA is involved in the control of prostate-cancer cell survival and modulates androgen receptor signaling. BMC cancer.

[39] Segawa T, Nau ME, Xu LL, Chilukuri RN, Makarem M, Zhang W (2002). Androgen-induced expression of endoplasmic reticulum (ER) stress response genes in prostate cancer cells. Oncogene.

[40] Tsui KH, Chang YL, Feng TH, Chang PL, Juang HH (2012). Glycoprotein transmembrane nmb: an androgen-downregulated gene attenuates cell invasion and tumorigenesis in prostate carcinoma cells. The Prostate.

[41] Labernadie A, Kato T, Brugués A, Serra-Picamal X, Derzsi S, Arwert E (2017). A mechanically active heterotypic E-cadherin/N-cadherin adhesion enables fibroblasts to drive cancer cell invasion. Nature cell biology.

[42] Wang M, Ren D, Guo W, Huang S, Wang Z, Li Q (2016). N-cadherin promotes epithelial-mesenchymal transition and cancer stem cell-like traits via ErbB signaling in prostate cancer cells. International journal of oncology.

[43] Cui Y, Yamada S (2013). N-cadherin dependent collective cell invasion of prostate cancer cells is regulated by the N-terminus of α-catenin. PloS one.

[44] Tanaka H, Kono E, Tran CP, Miyazaki H, Yamashiro J, Shimomura T (2010). Monoclonal antibody targeting of N-cadherin inhibits prostate cancer growth, metastasis and castration resistance. Nat Med.

[45] Cottard F, Asmane I, Erdmann E, Bergerat JP, Kurtz JE, Céraline J (2013). Constitutively active androgen receptor variants upregulate expression of mesenchymal markers in prostate cancer cells. PloS one.

[46] Angel P, Karin M (1991). The role of Jun, Fos and the AP-1 complex in cell-proliferation and transformation. Biochimica et biophysica acta.

[47] Cai C, Hsieh CL, Shemshedini L (2007). c-Jun has multiple enhancing activities in the novel cross talk between the androgen receptor and Ets variant gene 1 in prostate cancer. Molecular cancer research: MCR.

[48] Chen SY, Cai C, Fisher CJ, Zheng Z, Omwancha J, Hsieh CL (2006). c-Jun enhancement of androgen receptor transactivation is associated with prostate cancer cell proliferation. Oncogene.

[49] Lobaccaro JM, Poujol N, Terouanne B, Georget V, Fabre S, Lumbroso S (1999). Transcriptional interferences between normal or mutant androgen receptors and the activator protein 1-dissection of the androgen receptor functional domains. Endocrinology.

[50] Qiu Y, Tanaka T, Nawata H, Yanase T (2012). Dihydrotestosterone inhibits lectin-like oxidized-LDL receptor-1 expression in aortic endothelial cells via a NF-kappaB/AP-1-mediated mechanism. Endocrinology.

[51] Fronsdal K, Engedal N, Slagsvold T, Saatcioglu F (1998). CREB binding protein is a coactivator for the androgen receptor and mediates cross-talk with AP-1. The Journal of biological chemistry.

[52] Sato N, Sadar MD, Bruchovsky N, Saatcioglu F, Rennie PS, Sato S (1997). Androgenic induction of prostate-specific antigen gene is repressed by protein-protein interaction between the androgen receptor and AP-1/c-Jun in the human prostate cancer cell line LNCaP. The Journal of biological chemistry.

[53] Kallio PJ, Poukka H, Moilanen A, Janne OA, Palvimo JJ (1995). Androgen receptor-mediated transcriptional regulation in the absence of direct interaction with a specific DNA element. Molecular endocrinology (Baltimore, Md).

[54] Murtha PE, Zhu W, Zhang J, Zhang S, Young CY (1997). Effects of Ca++ mobilization on expression of androgen-regulated genes: interference with androgen receptor-mediated transactivation by AP-I proteins. The Prostate.

[55] Gazi MH, Gong A, Donkena KV, Young CY (2007). Sodium selenite inhibits interleukin-6-mediated androgen receptor activation in prostate cancer cells via upregulation of c-Jun. Clinica chimica acta; international journal of clinical chemistry.

[56] Hsu CC, Hu CD (2013). Transcriptional activity of c-Jun is critical for the suppression of AR function. Molecular and cellular endocrinology.

[57] Yuan H, Pan Y, Young CY (2004). Overexpression of c-Jun induced by quercetin and resverol inhibits the expression and function of the androgen receptor in human prostate cancer cells. Cancer letters.

[58] Lim SC, Jansson PJ, Assinder SJ, Maleki S, Richardson DR, Kovacevic Z (2020). Unique targeting of androgen-dependent and -independent AR signaling in prostate cancer to overcome androgen resistance. FASEB journal: official publication of the Federation of American Societies for Experimental Biology.

[59] Yuen RK, Chen B, Blair JD, Robinson WP, Nelson DM (2013). Hypoxia alters the epigenetic profile in cultured human placental trophoblasts. Epigenetics.

[60] Heiland DH, Ferrarese R, Claus R, Dai F, Masilamani AP, Kling E (2017). c-Jun-N-terminal phosphorylation regulates DNMT1 expression and genome wide methylation in gliomas. Oncotarget.

[61] Lin B, Hong H, Jiang X, Li C, Zhu S, Tang N (2018). c-Jun suppresses the expression of WNT inhibitory factor 1 through transcriptional regulation and interaction with DNA methyltransferase 1 in gallbladder cancer. Molecular medicine reports.

[62] Slack A, Pinard M, Araujo FD, Szyf M (2001). A novel regulatory element in the dnmt1 gene that responds to co-activation by Rb and c-Jun. Gene.

[63] Jaggi M, Nazemi T, Abrahams NA, Baker JJ, Galich A, Smith LM (2006). N-cadherin switching occurs in high Gleason grade prostate cancer. The Prostate.

[64] Chlenski A, Nakashiro K, Ketels KV, Korovaitseva GI, Oyasu R (2001). Androgen receptor expression in androgen-independent prostate cancer cell lines. The Prostate.

[65] Mitchell S, Abel P, Ware M, Stamp G, Lalani E (2000). Phenotypic and genotypic characterization of commonly used human prostatic cell lines. BJU international.

[66] van Bokhoven A, Varella-Garcia M, Korch C, Johannes WU, Smith EE, Miller HL (2003). Molecular characterization of human prostate carcinoma cell lines. The Prostate.

[67] Alimirah F, Chen J, Basrawala Z, Xin H, Choubey D (2006). DU-145 and PC-3 human prostate cancer cell lines express androgen receptor: implications for the androgen receptor functions and regulation. FEBS letters.

[68] Culig Z, Klocker H, Eberle J, Kaspar F, Hobisch A, Cronauer MV (1993). DNA sequence of the androgen receptor in prostatic tumor cell lines and tissue specimens assessed by means of the polymerase chain reaction. The Prostate.

[69] Edelstein RA, Carr MC, Caesar R, Young M, Atala A, Freeman MR (1994). Detection of human androgen receptor mRNA expression abnormalities by competitive PCR. DNA and cell biology.

[70] Tilley WD, Bentel JM, Aspinall JO, Hall RE, Horsfall DJ (1995). Evidence for a novel mechanism of androgen resistance in the human prostate cancer cell line, PC-3. Steroids.

[71] Jarrard DF, Kinoshita H, Shi Y, Sandefur C, Hoff D, Meisner LF (1998). Methylation of the androgen receptor promoter CpG island is associated with loss of androgen receptor expression in prostate cancer cells. Cancer research.

[72] Buchanan G, Craft PS, Yang M, Cheong A, Prescott J, Jia L (2004). PC-3 cells with enhanced androgen receptor signaling: a model for clonal selection in prostate cancer. The Prostate.

[73] Kandil S, Lee KY, Davies L, Rizzo SA, Dart DA, Westwell AD (2019). Discovery of deshydroxy bicalutamide derivatives as androgen receptor antagonists. European journal of medicinal chemistry.

[74] Sharma V, Kumar L, Mohanty SK, Maikhuri JP, Rajender S, Gupta G (2016). Sensitization of androgen refractory prostate cancer cells to anti-androgens through re-expression of epigenetically repressed androgen receptor - Synergistic action of quercetin and curcumin. Molecular and cellular endocrinology.

[75] Lunnon K, Mill J (2013). Epigenetic studies in Alzheimer's disease: current findings, caveats, and considerations for future studies. American journal of medical genetics Part B, Neuropsychiatric genetics: the official publication of the International Society of Psychiatric Genetics.

[76] Valente S, Liu Y, Schnekenburger M, Zwergel C, Cosconati S, Gros C (2014). Selective non-nucleoside inhibitors of human DNA methyltransferases active in cancer including in cancer stem cells. Journal of medicinal chemistry.

[77] Tinzl M, Chen B, Chen SY, Semenas J, Abrahamsson PA, Dizeyi N (2013). Interaction between c-jun and androgen receptor determines the outcome of taxane therapy in castration resistant prostate cancer. PloS one.

[78] Eggener SE, Scardino PT, Walsh PC, Han M, Partin AW, Trock BJ (2011). Predicting 15-year prostate cancer specific mortality after radical prostatectomy. The Journal of urology.

[79] Kozminski MA, Tomlins S, Cole A, Singhal U, Lu L, Skolarus TA (2016). Standardizing the definition of adverse pathology for lower risk men undergoing radical prostatectomy. Urologic oncology.

