# Supplementary material for: The role of N-cadherin/c-Jun/NDRG1 axis in the progression of prostate cancer
Source: Int J Biol Sci. 2021 Jul 25;17(13):3288–304. doi: 10.7150/ijbs.63300 (PMC8416735; doi:10.7150/ijbs.63300)
Supplement: Supplementary file 1 — Supplementary figure and tables. [file ijbsv17p3288s1.pdf]

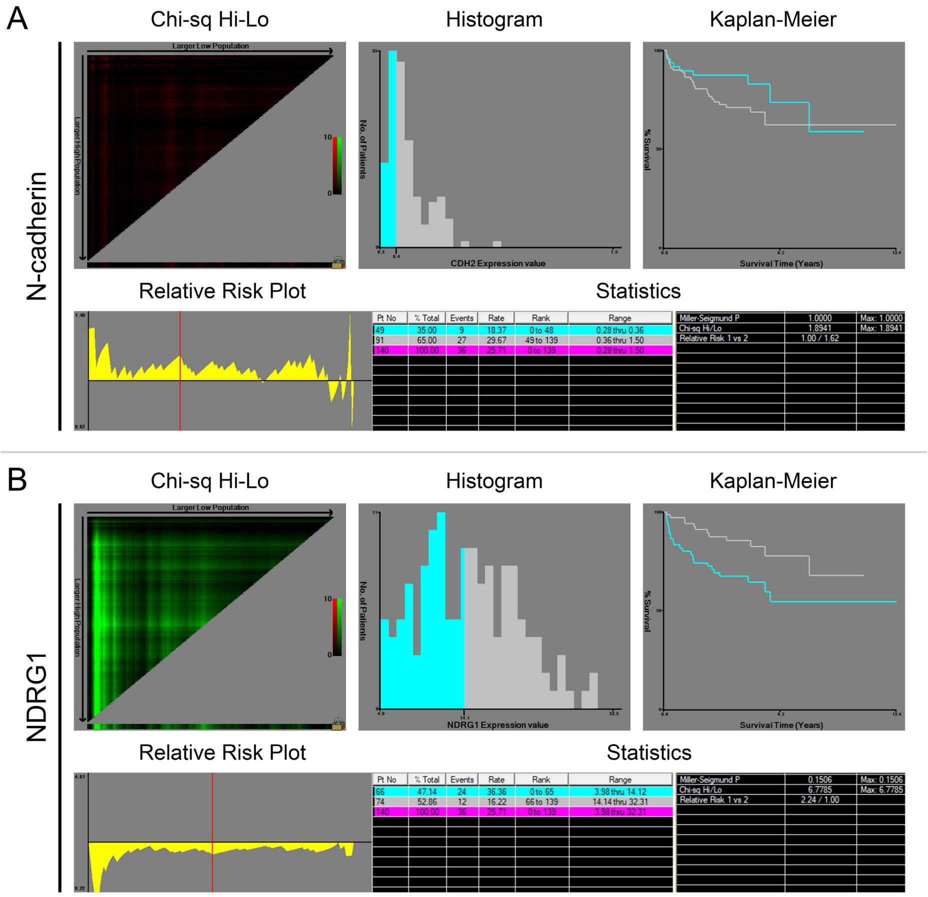

Supplementary Fig. 1.

Identification of the optimal cutoff values of N-cadherin (A) and NDRG1 (B) via X-tile analysis.

Supplementary Table 1. Information of gene regulation related sequences.

Name	Sequences		Vector
si-NDRG1	AACCUGCUACAACCCCCUCTT<br>sence (5'-3')	GAGGGGGUUGUAGCAGGUUTT<br>antisence (5'-3')	-
si-c-Jun	CAAACCUCAGCAACUUCAATT<br>sence (5'-3')	UUGAAGUUGCUGAGGUUUGTT<br>antisence (5'-3')	-
sh-CDH2	CCAGTGACTATTAAGAGAAAT<br>sence (5'-3')	ATTTCTCTTAATAGTCACTGG<br>antisence (5'-3')	hU6-MCS-Ubiquitin-EGFP-IRES-puromycin
oe-CDH2	NM_001792		Ubi-MCS-3FLAG-CBh-gcGFP-IRES-puromycin
oe-c-Jun	NM_002228		Ubi-MCS-3FLAG-SV40-EGFP-IRES-puromycin
oe-AR	NM_000044		Ubi-MCS-3FLAG-SV40-EGFP-IRES-puromycin

Supplementary Table 2. Relative Oligonucleotide primers.

Gene	Forward	Reverse
CDH2	CTTGTGCTGATGTTTGTGGTATGGATG	AGTCATAGTCCTGGTCTTCTTCTCCTC
AR	CTACATCAAGGAACTCGATCGT	CATGTGTGACTTGATTAGCAGG
NDRG1	GAAAAGCATTATTGGCATGGGA	CACAAGGGTTCACGTTGATAAG
18S	TTCGTATTGAGCCGCTAGA	CTTTCGCTCTGGTCCGTCTT
GAPDH	TGACTTCAACAGCGACACCCA	CACCCTGTTGCTGTAGCCAAA

Supplementary Table 3. Relative antibodies information.

Protein	Brand	NO.
AR	abcam	ab74272
NDRG1	CST	#9485
N-Cadherin	abcam	ab18203
E-cadherin	CST	#3195
ZEB1	CST	#3396
Slug	CST	#9585
Snail	CST	#3879
Vimentin	CST	#5741

CgA	abcam	ab15160
NSE	abcam	ab79757
Syn	abcam	ab32127
Ki-67	CST	#9449
Cleaved Caspase-3	CST	#9661
β-actin	CST	#3700

Supplementary Table 4. Co-IP and ChIP assay related information of antibodies.

**Protein**	**Brand**	**NO.**	**Host**
AR	abcam	ab74272	rabbit
c-Jun	abcam	ab31419	rabbit
DNMT1	abcam	ab13537	mouse
lgG	cst	#2729	rabbit
lgG	cst	#5873	mouse

Supplementary Table 5. ChIP assay related Oligonucleotide primers.

**Gene**	**Forward**	**Reverse**	**Region**
NDRG1-TRE (1)	TGGCCTGAGGACAATGACGG	GCCAGGTTGAGAAGCACTGC	-1153~-1064
NDRG1-ARE	GCCACCTGGGTAGCTTTGTA	AGAGGAGCCGCCAAATTAAA	-1051~-913
NDRG1-TRE (2)	GGGAACATGCAGGCCAATAGT	ACAAGCCCAGATGAGAGGATTC	-859~-727

Supplementary Table 6. Luciferase assay related information.

**Gene**	**Bank ID**	**Vector**
NDRG1-Promoter	NM_006096-promoter (-2000~-1)	MCS-firefly_Luciferase
NDRG1-Promoter (without ARE)	NM_006096-promoter (-2000~-985; -951~-1)	MCS-firefly_Luciferase
NDRG1-Promoter	NM_006096-promoter	MCS-firefly_Luciferase

(without TRE (1))	(-2000~-1125; -1117~-1)	
NDRG1-Promoter (without TRE (2))	NM_006096-promoter (-2000~-790; -782~-1)	MCS-firefly_Luciferase
NDRG1-Promoter (without TRE (1) and ARE)	NM_006096-promoter (-2000~-1125; -1117~-985; -951~-1)	MCS-firefly_Luciferase
NDRG1-Promoter (without TRE (1) and TRE (2))	NM_006096-promoter (-2000~-1125; -1117~-790; -782~-1)	MCS-firefly_Luciferase
NDRG1-Promoter (without ARE and TRE (2))	NM_006096-promoter (-2000~-985; -951~-790; -782~-1)	MCS-firefly_Luciferase
NDRG1-Promoter (without TRE (1), ARE, and TRE (2))	NM_006096-promoter (-2000~-1125; -1117~-985; -951~-790; -782~-1)	MCS-firefly_Luciferase
Renilla-Luciferase	-	TK promoter-Renilla_Luciferase

Supplementary Table 7. Pyrosequencing assay related primers.

**Primer name**	**Sequences (5'-3')**	**5' modification**	**Position of NDRG1 promoter**	**Analyzed CpG**
NDRG1(1)-Forward	GGTTTTGGGTTTAGTGGTAAAT		-1190~-1169	CpG (-1164) and CpG (-1136)
NDRG1(1)-Reverse	ACAAAACTACCCAAATAACTCCTA	5'-Biotin	-1033~-1056	
NDRG1(1)-Sequencing	TGGGTTTAGTGGTAAATT		-1185~-1168	
NDRG1(2)-Forward	GGTAGGTGGTTTGAGGATAAT		-1159~-1139	CpG (-1059)
NDRG1(2)-	ACAAAACTACCCAAATAACTCCTA	5'-Biotin	-1033~-1056	

Reverse				
NDRG1(2)-Sequencing	ATGTAGTGTTTTTTAATTTGGT		-1085~-1064	
NDRG1(3)-Forward	TGGTTTGTATGTTTTTATTTGGTTAAAGA		-844~-872	
NDRG1(3)-Reverse	ACCCCCTAAAATTCTAATTAAACTATTC	5'-Biotin	-1005~-978	CpG (-922)
NDRG1(3)-Sequencing	GTATATGAGAAGTTTTAGAGGA		-897~-918	
NDRG1(4)-Forward	TTTTTGGTTAGATGGGAATATGTAGGT		-872~-846	
NDRG1(4)-Reverse	CCCTTCCTATTCTAATTTCCTAAACTTTCA	5'-Biotin	-753~-782	CpG (-836) and CpG (-810)
NDRG1(4)-Sequencing	ATGGGAATATGTAGGTTAAT		-861~-842	
